# Biomolecular insights into North African-related ancestry, mobility and diet in eleventh-century Al-Andalus

**DOI:** 10.1038/s41598-021-95996-3

**Published:** 2021-09-13

**Authors:** Marina Silva, Gonzalo Oteo-García, Rui Martiniano, João Guimarães, Matthew von Tersch, Ali Madour, Tarek Shoeib, Alessandro Fichera, Pierre Justeau, M. George B. Foody, Krista McGrath, Amparo Barrachina, Vicente Palomar, Katharina Dulias, Bobby Yau, Francesca Gandini, Douglas J. Clarke, Alexandra Rosa, António Brehm, Antònia Flaquer, Teresa Rito, Anna Olivieri, Alessandro Achilli, Antonio Torroni, Alberto Gómez-Carballa, Antonio Salas, Jaroslaw Bryk, Peter W. Ditchfield, Michelle Alexander, Maria Pala, Pedro A. Soares, Ceiridwen J. Edwards, Martin B. Richards

**Affiliations:** 1 Department of Biological and Geographical Sciences, School of Applied Sciences, University of Huddersfield, Queensgate, Huddersfield, HD1 3DH UK; 2 Department of Genetics, University of Cambridge, Downing Street, Cambridge, CB2 3EH UK; 3 School of Biological and Environmental Sciences, Liverpool John Moores University, Liverpool, L3 3AF UK; 4 Department of Biology, CBMA (Centre of Molecular and Environmental Biology), University of Minho, Campus de Gualtar, 4710-057 Braga, Portugal; 5 BioArCh, Department of Archaeology, University of York, York, UK; 6 Department of Forensic Science, Faculty of Biomedical Science, University of Benghazi, P.O. Box: 1308, Benghazi, Libya; 7 Department of Prehistory and Institute of Environmental Science and Technology (ICTA), Universitat Autònoma de Barcelona, 08193 Bellaterra, Spain; 8 Servei d’Investigacions Arqueològiques i Prehistòriques - Museu Belles Arts de Castelló, Av. Germans Bou, 28, 12003 Castellón, Spain; 9 Museo Municipal de Arqueología y Etnología de Segorbe, Calle Colón, 98, 12400 Segorbe, Castellón Spain; 10 Institut für Geosysteme und Bioindikation, Technische Universität Braunschweig, Langer Kamp 19c, 38106 Braunschweig, Germany; 11 Faculty of Life Sciences, University of Madeira, Campus of Penteada, 9000-390 Funchal, Portugal; 12 Human Genetics Laboratory, University of Madeira, Campus of Penteada, 9000-390 Funchal, Portugal; 13 Institute for Medical Information Processing, Biometry and Epidemiology - IBE, LMU University, Munich, Germany; 14 Life and Health Sciences Research Institute (ICVS), School of Medicine, University of Minho, 4710-057 Braga, Portugal; 15 ICVS/3B’s, PT Government Associate Laboratory, 4710-057 Braga, Portugal; 16 Dipartimento di Biologia e Biotecnologie “L. Spallanzani, Università di Pavia, 27100 Pavia, Italy; 17 Grupo de Investigacion en Genetica, Vacunas, Infecciones y Pediatria (GENVIP), Hospital Clínico Universitario and Universidade de Santiago de Compostela, Galicia, Spain; 18 GenPoB Research Group, Instituto de Investigación Sanitaria (IDIS), Hospital Clínico Universitario de Santiago (SERGAS), 15706 Galicia, Spain; 19 Unidade de Xenética, Instituto de Ciencias Forenses (INCIFOR), Facultade de Medicina, Universidade de Santiago de Compostela, Galicia, Spain; 20 School of Archaeology, University of Oxford, 1 South Parks Road, Oxford, OX1 3TG UK; 21 Institute of Science and Innovation for Bio-Sustainability (IB-S), University of Minho, Campus de Gualtar, 4710-057 Braga, Portugal; 22 Present Address: Ancient Genomics Laboratory, The Francis Crick Institute, London, UK

**Keywords:** Anthropology, Archaeology, Evolutionary genetics, Population genetics

## Abstract

Historical records document medieval immigration from North Africa to Iberia to create Islamic al-Andalus. Here, we present a low-coverage genome of an eleventh century CE man buried in an Islamic necropolis in Segorbe, near Valencia, Spain. Uniparental lineages indicate North African ancestry, but at the autosomal level he displays a mosaic of North African and European-like ancestries, distinct from any present-day population. Altogether, the genome-wide evidence, stable isotope results and the age of the burial indicate that his ancestry was ultimately a result of admixture between recently arrived Amazigh people (Berbers) and the population inhabiting the Peninsula prior to the Islamic conquest. We detect differences between our sample and a previously published group of contemporary individuals from Valencia, exemplifying how detailed, small-scale aDNA studies can illuminate fine-grained regional and temporal differences. His genome demonstrates how ancient DNA studies can capture portraits of past genetic variation that have been erased by later demographic shifts—in this case, most likely the seventeenth century CE expulsion of formerly Islamic communities as tolerance dissipated following the *Reconquista* by the Catholic kingdoms of the north.

## Introduction

The location of Iberia, bridging the Mediterranean and the Atlantic, and its proximity to Africa, has allowed contacts with populations of distinct ancestries over time, making the Peninsula a genetic and cultural crossroads. There is both archaeological and direct genetic evidence of contacts between Iberia and North African populations since at least the Late Neolithic^1–6^, and possibly as early as the postglacial period^7,8^. Prehistoric populations have been the focus of most of the ancient DNA (aDNA) work published on Iberia so far, including the study of Mesolithic individuals^9,10^, the impact of Neolithic dispersals^11,12^, and the incursions of individuals with Steppe-related ancestry at the time of the transition from the Chalcolithic to the Bronze Age^5,6,13–15^.

aDNA researchers have recently begun to explore in detail historical intervals of known population movements^6^. Although Iberia intensified contacts with North Africa through Phoenician traders, Carthaginians and Roman conquerors^16^, North-African genetic contribution seems to have been restricted to southern populations until the eight century CE^6^. It is only with the Islamic conquest of Iberia in 711 CE that records start pointing towards a substantial influx of people from North Africa, involving the culturally and genetically differentiated Arab and Amazigh (Berber) peoples^17,18^. Attempts have been made to estimate their contribution to the genetic landscape of medieval Iberia using modern genomes, revealing a faint southwest-northeast pattern of decreasing North African-related ancestry^19,20^, which have recently been confirmed by means of aDNA analysis^6^.

Although Arabs were the urban and political elite during the Umayyad Caliphate, ruling from 711 CE until the end of the Caliphate of Cordoba in 1031 CE, they are thought to have been a minority amongst the new settlers. Berbers formed the bulk of the army who first seized Visigothic Spain in the eighth century CE^21^. Berbers had converted to Islam as a result of the Arab conquest of North Africa in the preceding century and embarked in a slow and complex process of Arabisation that lasted centuries. However, they were far from culturally homogeneous; a deep division existed between nomadic and sedentary Berber groups, and it was the latter who first settled in the rural areas of Spain^18^. Although Berber numbers in Iberia were likely larger than those of the Arabs, they initially wielded no significant political power, but this changed during the eleventh–thirteenth centuries CE with the establishment of the Almoravid and Almohad Berber empires^18^.

After the southwards military expansion of the Catholic kingdoms ended in 1492, a large population of *Moriscos* (Muslims forcibly converted to Christianity) persisted in East Iberia (previously Sharq al-Andalus) until 1609 CE, when at least one third of the populace was forcibly expelled by the Spanish Crown and relocated to North Africa^22^. Historical documentation suggests that the population of the eastern Mediterranean provinces of Castellón, Valencia, Alicante, and—to a lesser degree—Murcia and parts of Andalusia (Almeria and Granada), was greatly reduced, with subsequent resettlement from Aragon, Catalonia and Navarre to avoid economic and demographic collapse^23^. Many surnames currently widespread in the Valencian region are geographically structured and reflect their provenance from the colonizing regions (data from Spanish National Institute of Statistics, 2017; Supplementary Fig. S1). The hinterland was mostly repopulated by non-Catalan-speaking Aragonese, whereas the main coastal cities concentrated more Catalan-speakers from Catalonia^23^. This divide is thought to be still reflected in the genomic data today^20^. Thus, most of the existing genetic variation from the preceding eastern Iberian populations and the North African genetic variation potentially brought during Islamic rule had most likely disappeared by the late seventeenth century CE^20^, especially in the Valencian region^24^. Therefore, DNA from archaeological remains can provide an important tool to understand the demographic dynamics of the Islamic period in East Iberia^25^.

Here, we sequenced the genome of an individual (UE2298/MS060) who was buried in the Islamic *maqbara* (necropolis) of Plaza del Almudín in the city of Segorbe (province of Castellón, Comunidad Valenciana, Spain) (Supplementary Fig. S2). He was dubbed as “the Giant” by the archaeologists responsible for the excavation (here referred to as the “Segorbe Giant”), due to his unusual height (184–190 cm) compared with the other individuals found in the site (Barrachina 2004) (Supplementary Methods). Osteological assessment suggests that he had African ancestry, and he was postulated to be of possible Berber origin^26,27^. Although his uniparental lineages point to North African ancestry, at the autosomal level he displays both North African and European-related ancestries. The genetic analyses show differences in relation to contemporary individuals from Valencia^6^ and highlight the contribution of admixture between people of North African origin and the populations inhabiting East Iberia prior to the Islamic period. We conducted a complementary analysis of stable isotopes on a total of thirteen individuals from the necropolis (Supplementary Table S1) to investigate mobility and diet patterns. We also generated more than 1000 new modern Iberian whole mitochondrial genomes to assess the potential impact of North African mitochondrial DNA (mtDNA) lineages in the modern Iberian maternal gene pool. As UE2298/MS060 belongs to mtDNA haplogroup U6, we also performed a detailed phylogeographic reanalysis of this haplogroup.

## Results

### Uniparental genetic background of the Segorbe Giant

**Figure 1:**
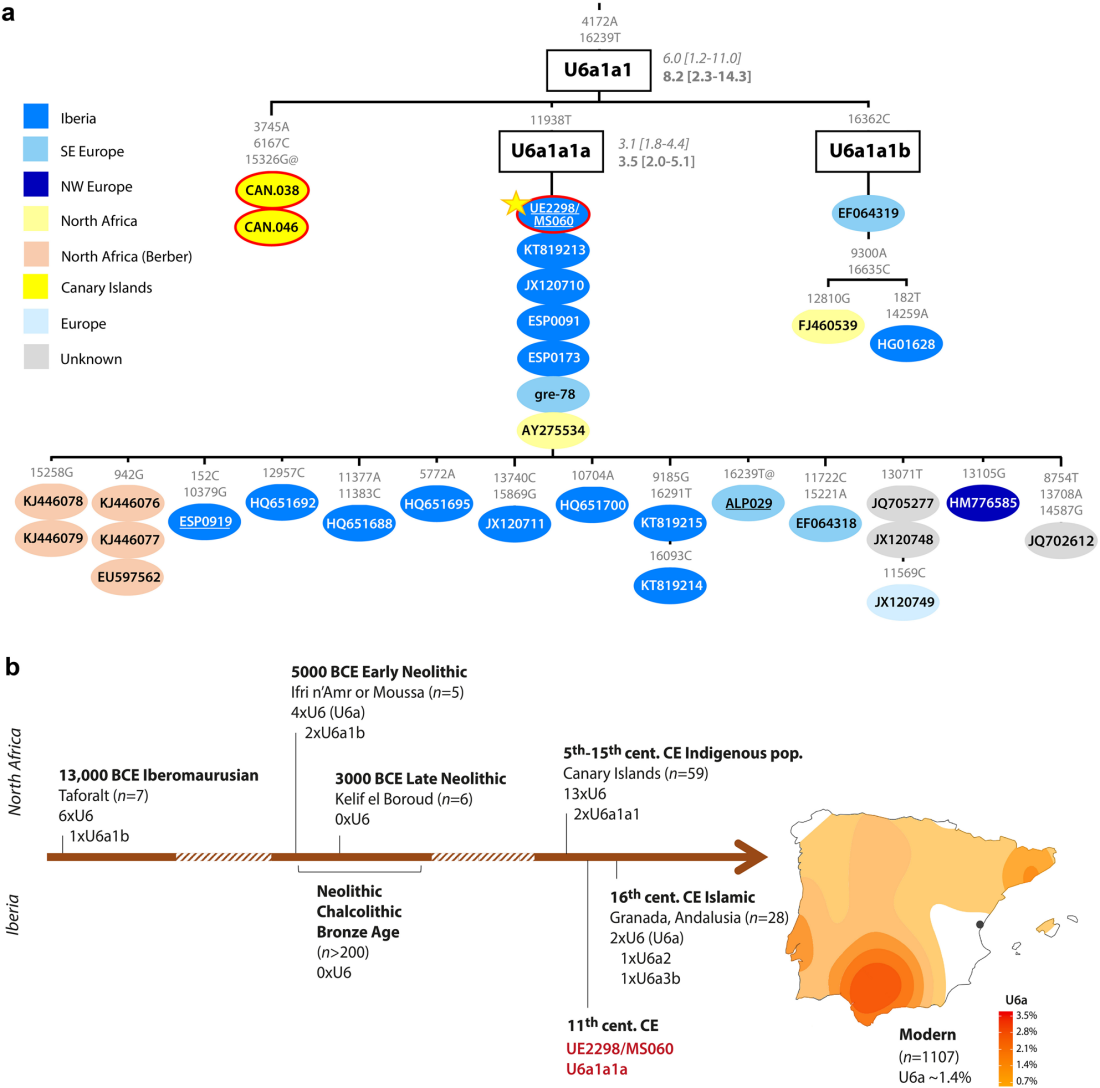
UE2298/MS060 maternal lineage. (**a**) Phylogenetic tree of mtDNA lineage U6a1a1. ρ and maximum-likelihood (ML) node age estimates (in ka) shown on the branches (in italics and in bold, respectively); sequences are coloured according to geography, with ancient sequences circled in red (position of UE2298/MS060 sequence is indicated by the star); underlined samples are newly reported; mutations relative to rCRS are indicated on the branches. The complete and more detailed tree for haplogroup U6 is shown in Supplementary Fig. S4. Details of the sequences used to build the tree are in Supplementary Table S4. (**b**) Timeline showing occurrence of haplogroup U6 in the archaeological record of North Africa and Iberia through time^2,6,13–15,32–35^, and a map of the frequency distribution of U6a in present-day Iberia, with a point indicating the location of Segorbe city. Density maps of additional mtDNA lineages are shown in Supplementary Fig. S5.

We confirmed that the individual was genetically male (*R*_Y_ > 0.077; Supplementary Fig. S3), and both his uniparental markers point towards North African origins (Supplementary Table S2). He belongs to mtDNA haplogroup U6a1a1a (nomenclature according to Hernández et al.^28^). Although U6 in general, and U6a in particular, is present in higher frequencies in North and West Africa^29,30^, the complete mitochondrial genome dataset currently available is heavily biased towards Europe, and U6a1a1a, which dates to 3.5 thousand years ago (ka) (maximum-likelihood node estimation based on modern variation), appears to have a more southern European distribution (Fig. 1a; Supplementary Fig. S4). However, in our Iberian mitogenome dataset, U6a1a1a occurs only at 0.3%, whereas the HVS-I (hypervariable segment I) subclade U6a1a1, defined by a transition variant at position 16239, which nests U6a1a1a, is found at ~ 14% in Algerian Mozabite Berbers^31^.

Haplogroup U6a1 has been found in Moroccan Iberomaurusian remains dating to 14–15 ka^32^, as well as in Early Neolithic Morocco (*i.e.* the pre-agricultural Holocene)^2^ (Fig. 1b). Although U6 lineages have been retrieved from sixteenth century CE Islamic burials in Granada (Andalusia)^6^, to our knowledge, UE2298/MS060 (dating to the eleventh century CE) is the earliest documented finding of a U6 lineage in Iberia. Based on the results of our newly generated Iberian mitochondrial dataset (*n* = 1104: 1008 sequences from mainland Spain and the Balearic Islands, plus 96 from mainland Portugal), U6a can be found at a frequency of 1.6% in modern mainland Iberian populations, with a peak of 3.6% in the south of Spain (Fig. 1b). This pattern contrasts with most mitochondrial lineages today in Iberia, although a peak of frequency in the south of the Peninsula is also observed for typically sub-Saharan African L lineages (but not for the predominantly northeast African haplogroup M1^36^) (Supplementary Fig. S5; Supplementary Table S5). UE2298/MS060 falls outside the modern geographic distribution of U6 lineages in Spain, suggesting that the present distribution might not reflect the medieval distribution of this haplogroup. A detailed phylogeographic analysis of U6 can be found in Supplementary Note 1.

**Figure 2:**
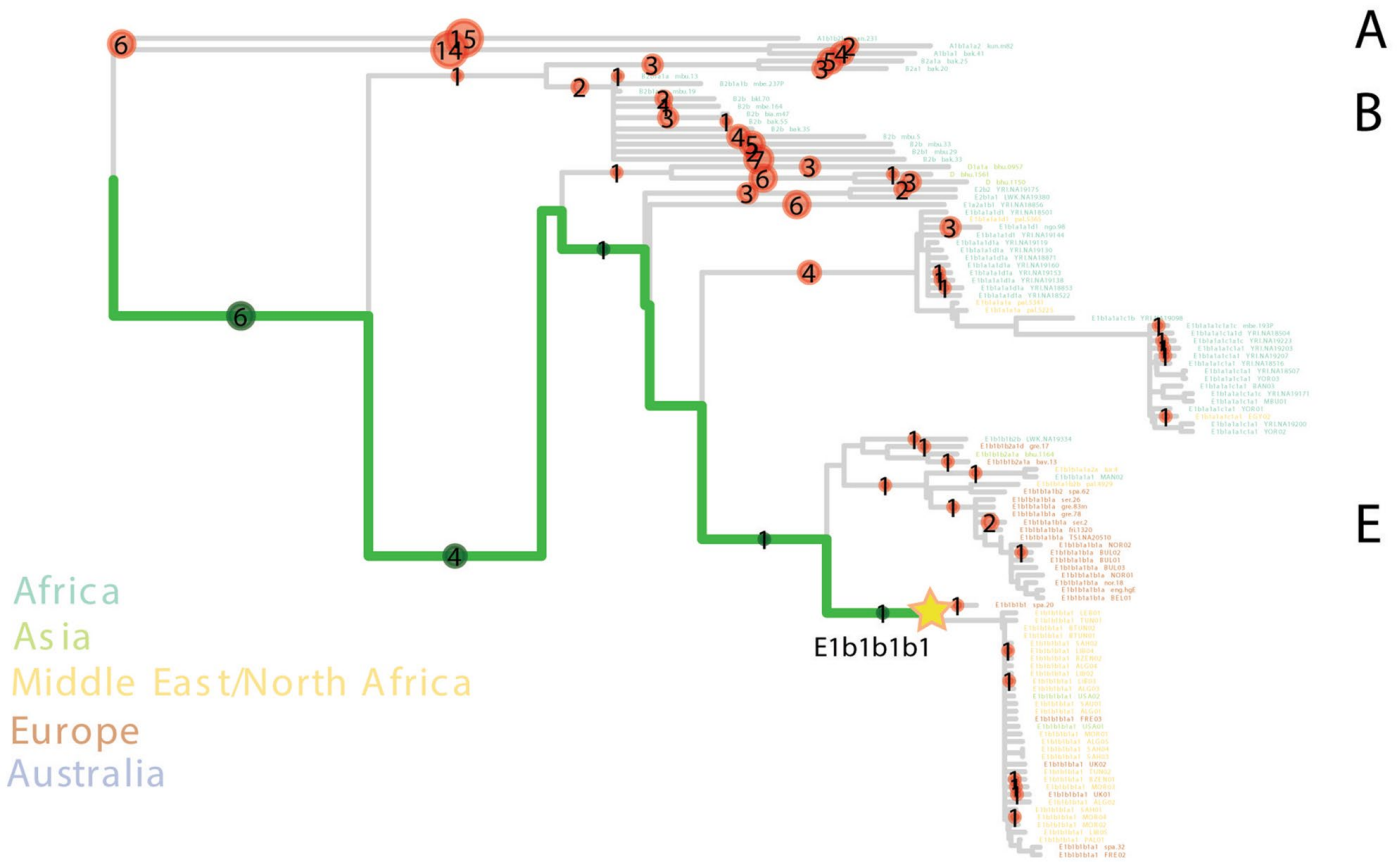
PathPhynder tree showing the position of UE2298/MS060 paternal lineage. Neighbour-joining phylogenetic tree estimated with 256 Y-chromosome sequences from worldwide populations^45,46^. Coloured circles indicate the number of derived (green) or ancestral (red) branch defining markers identified in the ancient individual. The branches coloured in green indicate the path with greatest support for the inclusion of UE2298/MS060 within a clade containing present-day Spanish, Near Eastern and North African individuals belonging to the E–M310 (E1b1b1b1) Y-chromosome lineage (indicated by the star). Label for haplogroups (A, B and E) provided on the right-hand side of the figure. The complete Y-chromosome tree is shown in Supplementary Fig. S7.

We assigned UE2298/MS060 to the Y-chromosome haplogroup E1b1b1b1 (E–M310) (Supplementary Table S2), dating to ~ 13.9 [12.1–15.7] ka (Yfull, v.6.06.15) and immediately basal to the clade nesting E–M81 (E1b1b1b1a) (Fig. 2; Supplementary Figs. S6 and S7). E1b1b is very frequent in contemporary North Africa and has been found in North African and Levantine remains^2,32,33,37^ (Supplementary Fig. S8). E–M81 (E1b1b1b1a), dating to ~ 2.8 ka (YFull, v.6.06.15), has been retrieved from early Islamic remains (seventh–eighth century CE) in southern France^38^, whereas the more derived E1b1b1b1a1 has been found in two individuals from an Islamic necropolis in the city of Valencia, dating to twelfth–thirteenth century CE^6^. E–M81 is today predominantly found in the Maghreb (where its average frequency is > 40%) and peaks in modern Berber populations, with frequencies reaching > 80%^39–41^, being almost fixed in some groups, such as the southern Moroccan Tachlhit-speakers^42^ and the Chenini–Douiret and Jradou from Tunisia^40^. In Europe, it is found mostly in Iberia and Sicily at frequencies < 5%^43^.

Given that there are no reads covering any of its diagnostic positions, we cannot exclude the possibility that UE2298/MS060 could belong to the E–M81 lineage (Supplementary Fig. S6). Using pathPhynder^44^ to investigate his Y-chromosomal affinity with present-day populations, UE2298/MS060 was positioned in a branch that harbours Iberian and North African E–M310-derived lineages, but with no support for membership to a more downstream lineage within this clade (Fig. 2; Supplementary Fig. S7).

### Genome-wide ancestry of the Segorbe Giant

**Figure 3:**
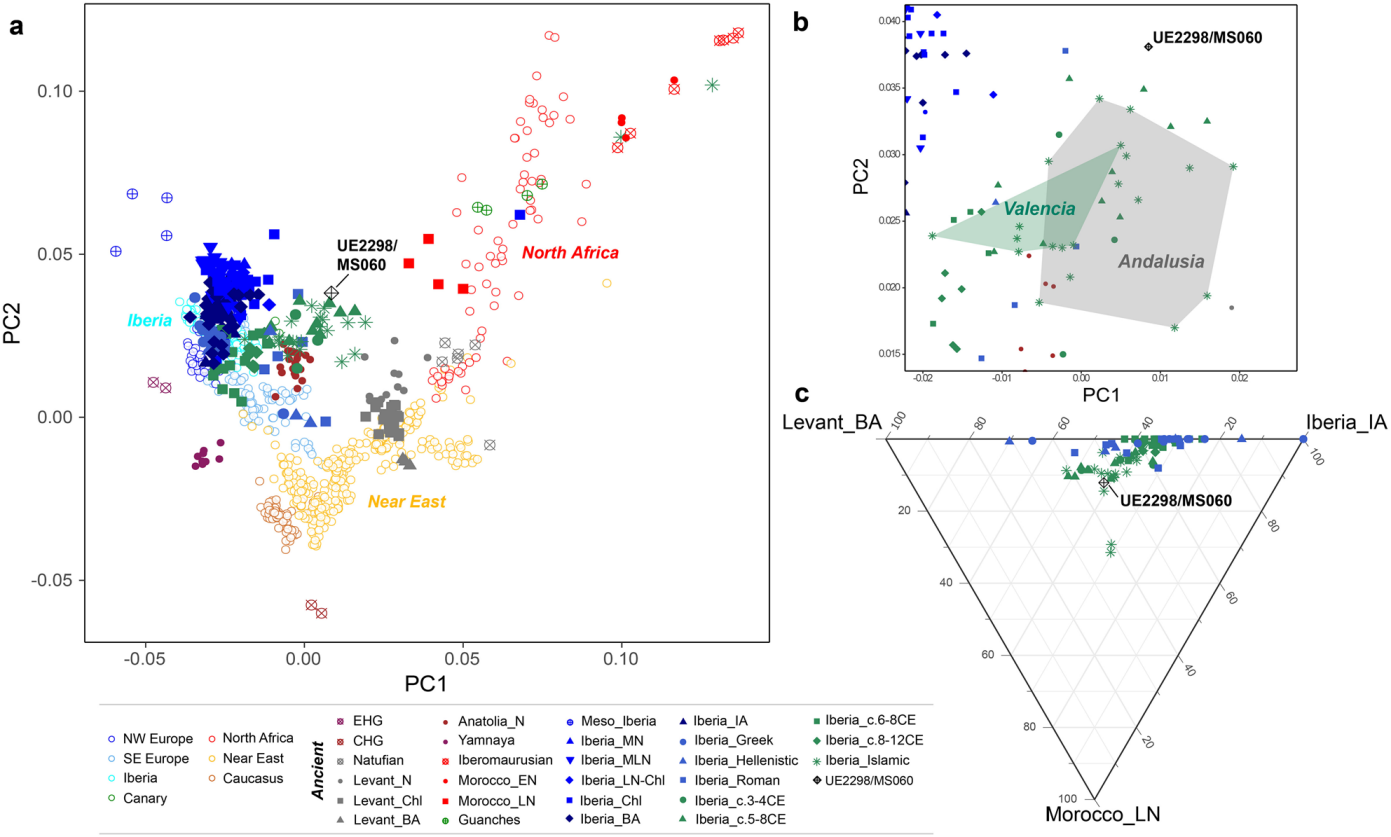
Overview of UE2298/MS060 autosomal ancestry. (**a**) PCA projecting 336 ancient samples on 702 modern individuals from North African, European, Near Eastern and Caucasian populations. (**b**) Zoom-in of PCA shown in (**a**) focussing on individuals from the Islamic period; individuals from Valencia and Andalusia (excluding two outliers that plot together with ancient North African individuals in (**a**)) within green and grey shapes, respectively. (**c**) Ternary plot showing supervised ADMIXTURE proportions (*K* = 3), using *Iberia_IA*, *Morocco_LN* and *Levant_BA* as reference populations. Abbreviations as follows: E/CHG, Eastern/Caucasus Hunter-Gatherers; Meso, Mesolithic; (E/M/L) N, (Early/Middle/Late) Neolithic; Chl, Chalcolithic; BA, Bronze Age; IA, Iron Age; c., centuries.

We investigated the autosomal ancestry of our ancient individual by calling ~ 74,200 autosomal SNPs (~ 72,300 when using a different approach to deal with post-mortem damage (Supplementary Table S2)). The PCA (Fig. 3a; Supplementary Fig. S9) shows that UE2298/MS060 occupies an intermediate position between present-day and ancient North African and Iberian populations in PC1, close to other Iberian Islamic individuals. Some differentiation between the Islamic individuals from Valencia and those from Andalusia is visible in the PCA, with the Andalusians mostly falling closer to North Africans and UE2298/MS060 falling outside both the Valencian and Andalusian clusters (Fig. 3b). However, this difference between UE2298/MS060 and the other Islamic individuals is not detected with ADMIXTURE in supervised mode (*K* = 3), using *Iberia_IA*, *Levant_BA* and *Morocco_LN/Guanches* as reference populations (following the findings in Olalde et al.^6^) (Fig. 3c; Supplementary Fig. S10).

**Figure 4:**
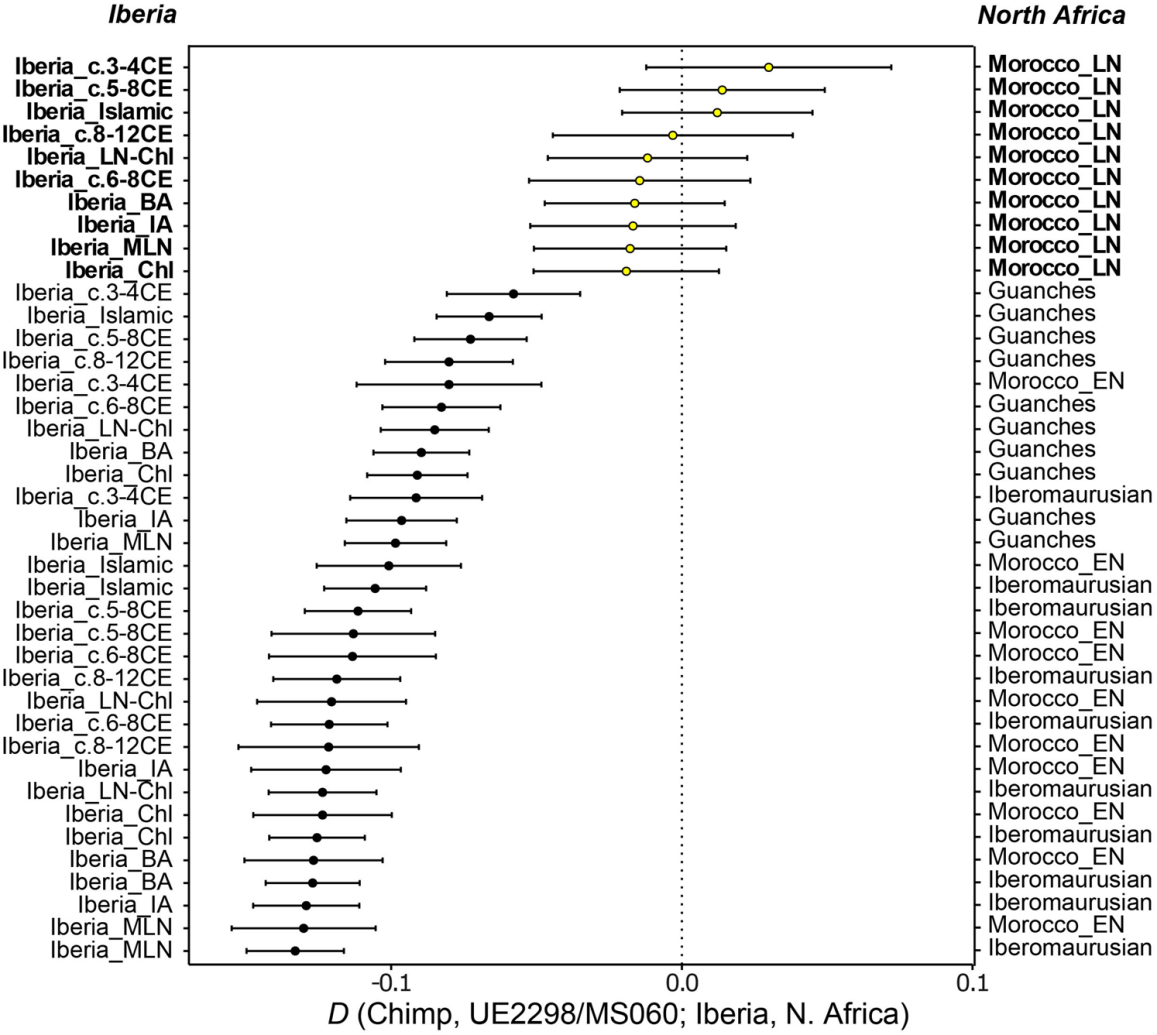
Detection of North African- and European-related ancestries in the genome of UE2298/MS060. *D(Chimp, UE2298/MS060; Iberian population, North African population)*. A significant negative *D-*value indicates that UE2298/MS060 shares more genetic drift with the Iberian population; a significant positive *D* indicates more shared drift with the North African population. Non-significant *D* indicates that UE2298/MS060 is symmetrically close to both populations tested (shown in yellow, with labels in bold). Error bars correspond to 2 standard errors. Detailed output can be found in Supplementary Table S7. Abbreviations as follows: (E/M/L)N, (Early/Middle/Late) Neolithic; Chl, Chalcolithic; BA, Bronze Age; IA, Iron Age; c., centuries.

Outgroup-*f3* runs using different outgroups (*Mbuti*, *Ju_hoan_North* and *Ust_Ishim*) consistently show a higher proportion of shared drift with Middle/Late Neolithic, Chalcolithic and Bronze Age Iberian populations, and with the Anatolian Neolithic (Supplementary Table S6), than with North African populations (although the proximity of North African groups, particularly Late Neolithic Morocco and the Guanches, to UE2298/MS060 changes when using Ust’-Ishim, a non-sub-Saharan African outgroup, suggesting that his genome may have some African-related ancestry). *D*-statistics consistently show UE2298/MS060 to be significantly closer to Iberian populations than to Iberomaurusians, Early Neolithic Morocco or the Guanches (Fig. 4; Supplementary Table S7). However, tests using Late Neolithic Morocco, in the form *D(outgroup, UE2298/MS060; Morocco_LN, Iberian population)*, consistently generated results close to zero and non-significant (|Z|-score < 3), which might be an indicator that a population genetically close to *Morocco_LN* contributed to the ancestry of UE2298/MS060 in similar proportions to an Iberian source. We note that we did not observe any major differences in the patterns observed for outgroup-*f3* and *D-*statistics using different approaches to minimise the effects of post-mortem damage (“mapDamage *—rescale*” and “soft-clipping”) (Supplementary Tables S6 and S7), but additional *qpAdm* models are accepted using “mapDamage *--rescale*” (Supplementary Tables S8 and S9).

We tested different *qpAdm* 1-way scenarios using different proximal Iberian sources as left populations. Models using populations from Andalusia (*Iberia_c.5-8CE* and *Iberia_c.3-4CE,* which already displayed North African-related ancestry^6^) are accepted (*p*-values: 0.092 and 0.343, respectively), whereas models using populations from Catalonia, in the northeast of the Peninsula, are rejected (*p*-value < 0.05) (Supplementary Table S8). However, considering the genetic heterogeneity in different regions of Iberia through time, and given the complex history of population interactions in Iberia during the first millennium CE^16,18^, it is unlikely that UE2298/MS060 descends directly from Andalusian Visigothic populations and therefore we also explored 2-way admixture scenarios. Notably, 1-way *qpAdm* analysis was consistent with UE2298/MS060 descending from *Islamic_Andalusia* (*p*-value = 0.327) but not from *Islamic_Valencia* (*p*-value = 0.0005), in line with the position of UE2298/MS060 in the PCA (Fig. 3b) and highlighting regional genetic differences during this period*.*

Alternatively, UE2298/MS060 could be modelled using 2-way combinations of distal and proximal Iberian populations (showing varied proportions of North-African related ancestry^6^) and either the *Guanches* or *Morocco_LN* (Table 1; Supplementary Table S9). *D*-statistics comparing these two North African populations indicate that UE2298/MS060 is closer to *Morocco_LN* (|Z|> 3) (Supplementary Table S7) than to the *Guanches*.

**Table 1:** Accepted 2-way *qpAdm* admixture models with standard errors (SE) and *p*-values. Models accepted using both datasets (“mapDamage *--rescale*” and “soft-clipping”) are shown in italics.

Target	Left populations		Admixture proportions		SE	*p*-value
	Pop. 1	Pop. 2	Pop. 1	Pop. 2		
UE2298/MS060	Guanches	Iberia_Islamic	0.102	0.898	0.066	0.148617
*UE2298/MS060*	*Guanches*	*Iberia_c.8-12CE*	0.172	0.828	0.058	0.220096
UE2298/MS060	Guanches	Iberia_c.5-8CE	0.122	0.878	0.064	0.168943
UE2298/MS060	Guanches	Iberia_c.3-4CE	0.091	0.909	0.07	0.391308
UE2298/MS060	Guanches	Iberia_IA	0.349	0.651	0.048	0.078756
UE2298/MS060	Morocco_LN	Iberia_Islamic	0.235	0.765	0.149	0.146791
*UE2298/MS060*	*Morocco_LN*	*Iberia_c.8-12CE*	0.308	0.692	0.13	0.099908
UE2298/MS060	Morocco_LN	Iberia_c.6-8CE	0.593	0.407	0.059	0.053639
UE2298/MS060	Morocco_LN	Iberia_c.5-8CE	0.18	0.82	0.17	0.091952
UE2298/MS060	Morocco_LN	Iberia_c.3-4CE	0.094	0.906	0.23	0.287481

### Mobility in Islamic Segorbe

**Figure 5:**
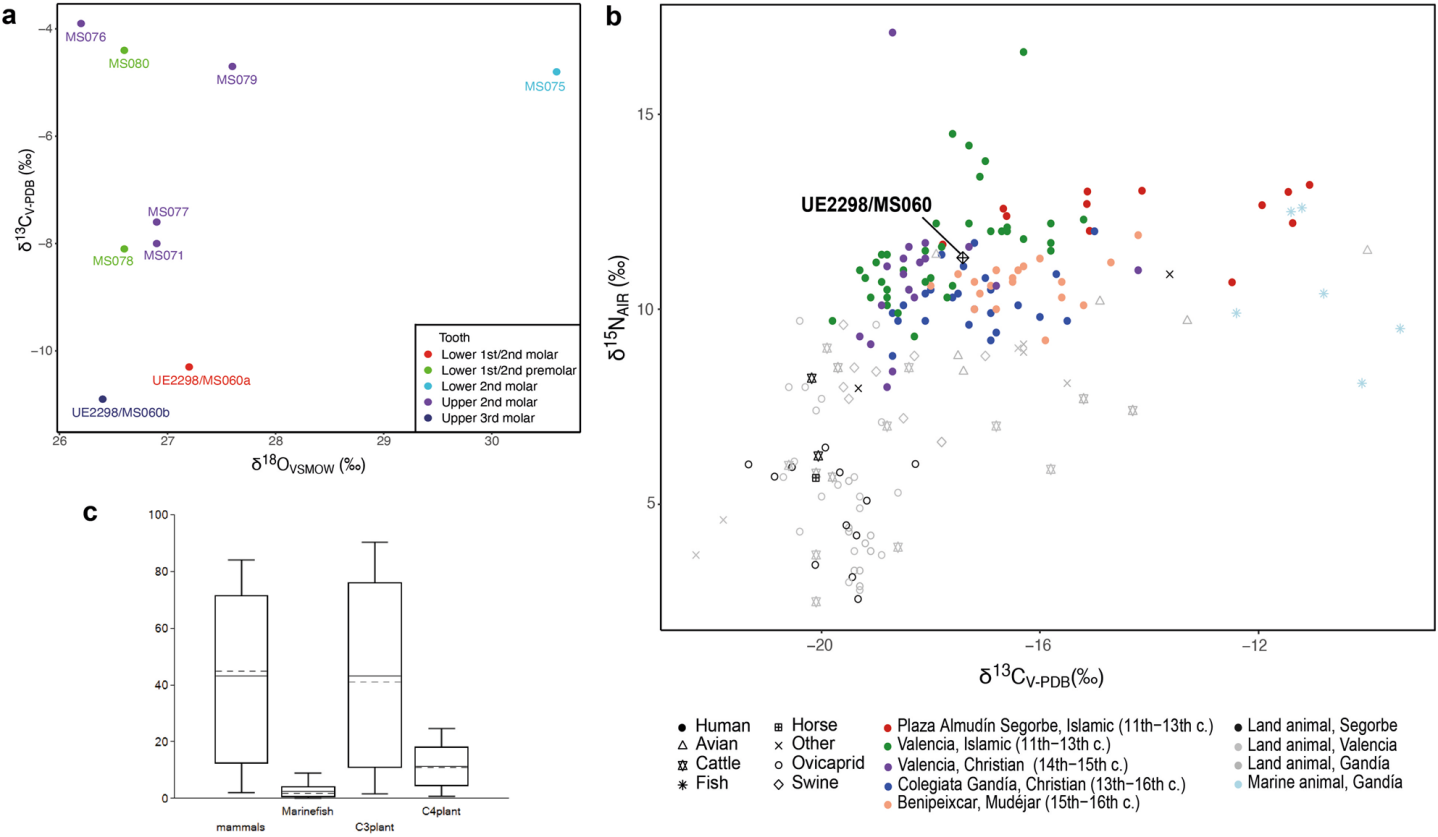
Mobility and diet in Islamic Segorbe. (**a**) Mobility isotopes (oxygen and carbon) for UE2298/MS060 and other individuals from Plaza del Almudín. (**b**) Dietary isotopes (carbon and nitrogen) from Plaza del Almudín compared to other medieval Islamic and Christian sites from Gandía and Valencia^51,52^. (**c**) FRUITS model for UE2298/MS060; models for other individuals can be found in Supplementary Fig. S11.

In order to assess whether or not UE2298/MS060 was likely to have spent their childhood in the local region, we performed stable oxygen analysis on eight individuals from Plaza del Almudín. Tooth enamel carbonate data is presented in Supplementary Table S10 and plotted in Fig. 5a. The δ^18^O_VSMOW_ values for the Segorbe population (excluding outlier MS075) range from 26.2 to 27.6‰ (range = 1.4‰, *n* = 7), with a mean of 26.8 ± 0.5‰ (1σ). The converted δ^18^O_dw_ values (mean -6.0‰, excluding MS075) fit with the meteoric water values for eastern Iberian coast. The δ^18^O_VSMOW_ values from both teeth sampled from UE2298/MS060 are consistent with the rest of the population and the small difference in values between the different molars (M1/M2 and M3) provide no indication of movement between early childhood and adolescence. Overall, there is no evidence that UE2298/MS060 was an immigrant in East Spain, on the basis of his oxygen values.

By contrast, one other individual reported here (MS075) seems to be an outlier (δ^18^O_VSMOW_ = 30.6; > 1.5 times the interquartile range above quartile 3)^47^, and possibly a migrant from a warmer climate, with a δ^18^O_dw_ value similar to Africa or the Near East^48^. Detailed results and discussion of oxygen analysis can be found in Supplementary Note 2.

### Diet patterns in Islamic Segorbe

The values for δ^15^N and δ^13^C dietary isotopes in the Islamic necropolis of Plaza del Almudín range between 10.7 to 13.2‰ and from –17.8 to –11‰, respectively, for the 13 individuals studied (Fig. 5b; Supplementary Table S11). UE2298/MS060 has a δ^15^N value of 11.3‰ and a δ^13^C value of –17.4‰, showing lower δ^15^N and a more negative δ^13^C than the majority of the humans sampled from this assemblage. Application of a Bayesian mixing model (BMM), FRUITS (Food Reconstruction Using Isotopic Transferred Signals)^49^, supports the observation that C_4_ plants likely played a substantial part in the diet of some individuals and that marine fish consumption was variable (Supplementary Fig. S11). UE2298/MS060 (Fig. 5c) seems to have consumed limited amounts of C_4_-plants (mean: 11.4 ± 6.5% or 4.8–17.9% of the diet) and marine protein (mean: 2.4 ± 2.4% or 0–4.8% of the diet) compared to the rest of the population analysed. On the other hand, he seems to have the highest levels of mammal and C_3_-plant consumption amongst the analysed individuals (Supplementary Fig. S11).

Individual MS075, identified as a possible migrant due to their oxygen value, displays the lowest probability (close to zero) of marine fish consumption amongst the individuals studied here (Supplementary Fig. S11), and shows signals of a mixed C_3_/C_4_ diet, which is also a possibility for Africa^50^. Detailed results and discussion of diet patterns inferred from individuals from the site of Plaza del Almudín can be found in Supplementary Note 2.

### Discussion

We analysed individual UE2298/MS060 excavated from the Islamic necropolis of Plaza del Almudín, in Segorbe, dating to the eleventh century CE. The archaeologists responsible for the excavation in 1999 considered this individual unusual due to his considerable height compared with other individuals found at the same site (despite periods of disease and/or malnutrition in childhood)^27^, and dubbed him the “Segorbe Giant”. The subsequent anthropological analysis suggested some African morphological features and a link was postulated to the Berber-speaking populations that settled in the region in medieval times^26,27^.

Analysis of the uniparental markers from UE2298/MS060 fits well with this assumption, pointing to an origin in the Maghreb, most likely from a Berber group. MtDNA lineage U6a is not only connected to modern Amazigh populations^30^, but has also been found in Moroccan remains associated with Iberomaurusian culture, and in the Moroccan Early Neolithic site of Ifri n’Amr or Moussa^2,32^ (Fig. 1b). He also carries the Y-chromosome E1b1b1b1 (E–M310) lineage. E1b1b is extremely common amongst extant North Africans and has been found in ancient North African and Levantine remains^2,32,33,37^ (Supplementary Fig. S7). Due to low coverage, we could only assign him to a basal position within E1b1b1b1, but it is possible that he may belong to a more derived subclade. One possibility would be E1b1b1b1a (E–M81), which is the most common haplogroup amongst modern Berber males today^42,53^, and has been linked to Islamic remains in southern France^38^. Another would be its descendant E1b1b1b1a1-M183 lineage, identified in three Guanche males, in two Islamic individuals from Granada, and in an earlier sixth century CE male from the Visigoth phase of Pla de l'Horta, in Catalonia^6,33^.

Although he carries both uniparental markers of North African origin, autosomal evidence paints a more complex picture. The individual is positioned in the PCA mid-way between modern/ancient Iberian populations, and Late Neolithic Moroccan, Guanches and modern North African individuals (Fig. 3a), and formal tests of admixture point to high proportions of Iberian-like ancestry (Fig. 4; Supplementary Table S7).

Considering the archaeological and historical records for this period in the region of Valencia, we envisage three possible scenarios to explain the observed ancestry in UE2298/MS060. One would be to assume that this individual is a direct migrant from North Africa (whose unique genetic composition has not yet been examined using aDNA), or derives from a population that moved into Iberia but retained its genetic identity. A second scenario is that he descends from pre-Islamic Iberian genetic diversity. Finally, the third scenario is that he is the result of admixture between Iberian and North African sources.

The first scenario would imply that pre-Islamic populations in North Africa would be genetically similar to UE2298/MS060 (or possibly to other contemporary individuals found in Spain^6^). The nearest temporal proxy available are the Guanches (from the seventh–eleventh centuries CE), who originated in the Maghreb but have been isolated in the Canary Islands since at least the early Iron Age. *D*-statistics, however, suggest that UE2298/MS060 is genetically closer to *Morocco_LN* than to the *Guanches* (Supplementary Table S7). In any case, *qpAdm* rejects the hypothesis that UE2298/MS060 directly descends from a population resembling either the *Guanches* or *Morocco_LN* (Supplementary Table S8). Additionally, the oxygen data for UE2298/MS060 (Supplementary Note 2) is consistent with someone who grew up in the region, and points towards low mobility between early childhood and adolescence. (In contrast, another individual from the same necropolis (MS075) does look non-local (Supplementary Note 2), possibly a migrant from a warmer climate outside the Mediterranean, with oxygen values similar to those of Africa or the Near East^48^). Nevertheless, one should note that aDNA sampling in North Africa is sparse and limited to a few individuals from very specific sites and periods, and we cannot rule out that a population with a similar genetic composition to that of UE2298/MS060 existed in the region around this period.

Although North African-related ancestry in present-day Spain is present at low values (typically ~ 3–8%), with a slight southwest-to-northeast decline^19,20^, increased African-related ancestry has been present in south Spain since the third century CE^6^. This North African influence is captured in our *qpAdm* analysis, with 1-way models using pre-Islamic Andalusian populations being accepted (Supplementary Table S8). However, it is unlikely that UE2298/MS060 descends directly from Andalusian Visigothic populations and ultimately these models, despite being statistically plausible, do not fully explain the ancestry of our individual. We note that there are no data available from or around the region of Valencia between the end of the Iron Age and the Islamic period, and post-Iron-Age genetic variation in Spain was most likely very heterogeneous across locations and centuries^6^. This heterogeneity is confirmed by our results showing that UE2298/MS060 forms a clade with *Islamic_Andalusia*, but not with *Islamic_Valencia* (Supplementary Table S8).

The third scenario would be that the genetic variation seen in UE2298/MS060 was a result of admixture between Amazigh people who migrated from North Africa to Iberia, and the local population inhabiting the Peninsula, at some point during either the Islamic conquest, the Caliphate period, or the Berber empires. This would explain UE2298/MS060's intermediate position in the PCA and ternary plot (supervised ADMIXTURE) (Fig. 3). *D*-statistics support this scenario, with tests comparing Morocco Late Neolithic and Iberian populations from different periods not showing him to be significantly closer to one or the other (Fig. 4; Supplementary Table S7). We show that UE2298/MS060 can be modelled as admixture between Iberian and North African sources (either the Guanches from the Canary Islands or Late Neolithic Moroccans) (Table 1). The fact that he still carried both uniparental markers of North African origin suggests that the admixture may have happened only a few generations before his time, coinciding with the zenith of Berber power, rather than earlier during the conquest, in agreement with admixture dates inferred from modern Iberian genomes from Aragon and Catalonia^20^. However, we cannot rule out assortative mating, allowing these uniparental markers to be retained for longer, or the possibility that these lineages were common in some Iberian populations before the Islamic period. The date of the burial (eleventh century CE)^27^ fits the historical narrative of Berber settlement in the region of Sharq al-Andalus^18^. Considering the genetic evidence, together with the stable isotope results and the historical accounts of intermarriage between local individuals and the North African newcomers, and in agreement with recent aDNA evidence from Iberia^6^, this third scenario seems the most plausible to explain the ancestry patterns seen in his genome.

Nevertheless, the original source populations are difficult to pinpoint. Due to lack of sampling in North Africa for this specific period and preceding centuries, the nearest proxies available for the North African source are the Guanches^33^ and the Late Neolithic Moroccan population from Kelif el Boroud site^2^. There is high differentiation between present-day North African populations and ancient North African individuals available to date (seen in PC3; Supplementary Fig. S9), which indicates that important population dynamics occurring after the Late Neolithic and/or Iron Age shaped extant genetic structure in the region. Modern North African populations show a signal of increased Levantine-related ancestry around the seventh century CE, as a result of movements from the Near East during the Islamic expansion into North Africa^17^; the impact of these movements was also seen in the Levant, as shown by the study of seventh–eighth century Islamic individuals in Syria^54^. Therefore, the North-African source of UE2298/MS060 might have already displayed this increased Near Eastern-related ancestry. Similarly, the population of Valencia in the immediately preceding centuries has yet to be studied.

A study in modern South Americans detected North African ancestry introduced at the early stages of European colonization^55^. The presence of individuals in medieval Spain with a genetic background similar to that of UE2298/MS060 would explain the source of this ancestry in America, suggesting that admixture with North Africans had a wider impact on medieval Spanish genetic variation, before virtually disappearing in the following centuries.

We found no U6 in our present-day whole-mtDNA dataset from the region of Valencia (*n* = 54), or in a larger previously published HVS-I database (*n* = 123)^56^. This absence might be an echo of the brutality of the decree of expulsion of *Moriscos* (Muslims forcibly converted to Christianity), which may have effectively erased the population carrying North African-related ancestry that lived in the region in the preceding centuries. They were replaced by settlers from regions further north with little North African-related ancestry^20^. This is in sharp contrast with regions of the Crown of Castilla, where historical sources claim there was better integration of the *Morisco* identity into the general population, and where no mass deportations were recorded: the frequency of U6, M1 and L lineages are higher in these regions today (present-day central and south Spain) (Fig. 1b; Supplementary Fig. S5). This pattern is also visible at the genome-wide level^20^.

This study emphasises the importance of immigration during the Islamic period. In contrast to Andalusia, the region of Valencia is not geographically close to the Maghreb, and was under Islamic rule for a shorter time, but nonetheless developed strong links with the Arab–Berber world during the Islamic period^57^. A contemporary individual, MS075, is evidence of continued movement during Berber rule (Supplementary Note 2).

UE2298/MS060 is a single, low-coverage sample and although the results cannot be extrapolated to the population as a whole, recently published results^6^ show a similar trend of admixture in Islamic Spain. The heterogeneity of genomic patterns that is now being uncovered by aDNA studies emphasises the need for much more detailed, high-resolution fine-scale studies. More individuals and a wider diversity of sites across the Peninsula should be studied to explore the population dynamics during the Islamic period in more detail and assess potential fine differences between geographical regions and periods, and between urban and rural societies.

## Methods

### Islamic Segorbe: aDNA and stable isotope analysis

We collected teeth from thirteen individuals from the medieval Islamic necropolis of Plaza del Almudín in Segorbe^27^ (province of Castellón, Spain) (Supplementary Fig. S2; Supplementary Table S1). Although the necropolis is dated to the eleventh–thirteenth centuries CE, the samples studied here come from a context dated to the eleventh century. We screened three individuals for aDNA, but only one, UE2298/MS060 (dubbed the “Segorbe Giant” due to his unusual height), excavated in 1999, yielded sufficient DNA for genomic analysis (Supplementary Fig. S2; Supplementary Table S2). We undertook stable isotope analyses on a total of thirteen individuals (including UE2298/MS060), and sixteen bone fragments from animals found in the site (although these might post-date the timeframe of the Islamic necropolis of Plaza del Almudín and belong instead to the later Christian context). All samples were collected from the Museo Municipal de Arqueología y Etnología de Segorbe, and permissions were agreed by the museum and granted by the Direccio General de Cultura i Patrimoni (Conselleria d’Educacio, Investigacio, Cultura i Esport de la Generalitat Valenciana).

We processed all the archaeological samples in clean rooms in the specialized Ancient DNA Facility at the University of Huddersfield. We sequenced one USER™-treated library on a tenth of an Illumina HiSeq4000 lane (100 cycles) to screen for endogenous aDNA content, and later sequenced three additional libraries (one of which was non-USER treated) in half an Illumina HiSeq4000 lane (100 cycles) (Macrogen, inc., Seoul, South Korea). We performed oxygen analysis and ZooMS (for taxonomic identification of the faunal assemblage) at the University of York, and dietary isotope analysis of carbon and nitrogen at the Research Laboratory for Archaeology, University of Oxford. Further details of ancient DNA, stable isotope and ZooMS analyses can be found in Supplementary Methods.

### Sequence data processing

We assessed raw read quality with FastQC v.0.11.5^58^, and merged paired-end reads and removed sequencing adapters using leeHom^59^. We mapped reads both to the human genome reference (hg19, modified to include rCRS (revised Cambridge Reference Sequence) instead of chrM) and to only the rCRS with BWA v.0.7.5a-r405^60^ *aln* (using the optimized settings for aDNA mapping^61^) and *samse*. We performed quality control of the alignment with QualiMap v.2.2^62^ and confirmed aDNA authenticity by checking contamination estimates (schmutzi^63^ and ANGSD^64^) and post-mortem damage patterns (Supplementary Fig. S12), as well as consistency in mtDNA haplogroup and sex assignment across all libraries. To avoid SNP miscalls due to post-mortem damage, we followed two approaches: (1) downscaling base quality of positions likely affected by post-mortem misincorporations using mapDamage^65^ *--rescale*; and (2) soft-clipping the terminal 3 base pairs of sequencing reads using the *trimBam* option in bamUtil package v. 1.0.14^66^, to control for potential reference bias resulting from downscaling base quality scores that could influence formal tests of admixture. We merged all libraries using picard *MERGESAM* (https://github.com/broadinstitute/picard). Detailed methods and parameters can be found in Supplementary Methods.

### Analysis of mtDNA and Y-chromosome variation

We used HaploGrep 2.0^67^ to classify mtDNA haplogroups. We performed Y-chromosome haplogroup classification using Yleaf^68^, and checked mutations against the ISOGG (International Society of Genetic Genealogy) SNP index (as of June 2018). We used pathPhynder^44^ (https://github.com/ruidlpm/pathPhynder) to investigate the affinity of individual UE2298/MS060 with present-day Y chromosomes^45,46^. More details on the analysis of uniparental markers of UE2298/MS060 can be found in Supplementary Methods.

### Autosomal DNA analysis

We called pseudo-haploid autosomal SNPs (Supplementary Table S2) against the 1240k SNP list for UE2298/MS060 (available at https://reich.hms.harvard.edu/) using samtools *mpileup* and *pileupCaller* (https://github.com/stschiff/sequenceTools). We used *convertf* and *mergeit* (both included in EIGENSOFT v.7.2.1 package^69^) to merge and convert files when necessary.

We compiled a dataset with ~ 1.2 M SNPs for analysis using only ancient samples. Published ancient samples were remapped to our reference and reanalysed alongside UE2298/MS060 to prevent possible batch effects due to differences in pipelines. Principal component analysis (PCA) of ~ 600 k autosomal SNPs was performed using smartpca (EIGENSOFT v.7.2.1) to project 336 ancient samples (Supplementary Table S3) on a selection of 702 modern individuals from North Africa, Europe, the Caucasus and the Near East^37^.

We filtered the ancient dataset for positions in linkage disequilibrium (LD) using the command *--indep-pairwise* (200, 25, 0.4) in PLINK v.1.07^70^. The LD pruned dataset (~ 450 k SNPs) was used to run ADMIXTURE v.1.3.0^71^ for post-Iron Age Iberian individuals (shown to display different levels of North African and Levantine-associated ancestries^6^) in supervised mode for *K* = 3 (with parameters: *--cv* and *--seed time*), using *Iberia_IA*, *Morocco_LN/Guanches* and *Levant_BA* as reference populations (Supplementary Table S3).

We added outgroups to the ~ 1.2 M SNP dataset for the formal tests of admixture (ADMIXTOOLS v.4.1^72^), and ran the tests in the two datasets (generated using “mapDamage *--rescale*” and “soft-clipping”). We examined patterns of allele sharing between UE2298/MS060 and present-day and ancient populations using outgroup-*f3* statistics, as implemented in *qp3Pop*, testing three outgroups (*Mbuti*, *Ju_hoan_North*, *Ust_Ishim*) to account for deeply divergent human ancestry. We computed *D*-statistics (using chimpanzee and Mbuti as outgroups) with *qpDstat* to untangle Iberian and North African-related contributions. For a more refined analysis, we ran a test with the formula *D(outgroup, UE2298/MS060; Islamic_Valencia, Islamic_Andalusia).* In order to investigate admixture proportions in the genome of UE2298/MS060, we ran *qpAdm* (ADMIXTOOLS v.4.1), using *allsnps: YES* and testing 1- and 2-way models. Following 2-way *qpAdm* results, we ran a *D*-statistics test in the form *D(outgroup, UE2298/MS060; Morocco_LN, Guanches)*. All plots were created with RStudio^73^. Detailed methods and parameters can be found in Supplementary Methods.

### Modern Iberian mtDNA dataset

We newly sequenced a total of 1126 mitogenomes from present-day Spain and Portugal (including samples assigned to insular territories, Melilla and Ceuta) with Illumina MiSeq paired-end sequencing (size of fragment: 150 bp) (Earlham Institute, Norwich Science Park, UK). A detailed description of the long-range PCR protocol, sequencing and data analysis can be found in Supplementary Methods.

### Phylogeographic analysis of mtDNA haplogroup U6

We performed a reassessment of phylogeographic patterns of mtDNA haplogroup U6 based on a total of 330 modern (35 of which are newly published here) and 32 ancient sequences (including UE2298/MS060) (Supplementary Table S4). Detailed description of the methods can be found in Supplementary Methods.

### Ethics statement

All archaeological samples were collected from the Museo Municipal de Arqueología y Etnología de Segorbe, and permissions were agreed by the museum and granted by the Direccio General de Cultura i Patrimoni (Conselleria d’Educacio, Investigacio, Cultura i Esport de la Generalitat Valenciana). For the present-day dataset written informed consent was obtained from all sample donors. The research was performed in accordance with the relevant guidelines and regulations and was approved by the University of Huddersfield’s School of Applied Sciences Ethics Committee, the Ethical Committee of the University of Santiago de Compostela, the Ethics Committee for Clinical Experimentation of the University of Pavia and the Ethics Committee of the University of Minho. Portuguese modern samples (PT-codes) were collected among army volunteers, upon approval of the Portuguese Army Chief of Staff, and were fully anonymized with the single purpose of use for population studies.

## Supplementary Information

Supplementary Information.

Supplementary Figure S4.

Supplementary Tables.

## Data Availability

Sequence data for UE2298/MS060 can be downloaded from the European Nucleotide Archive (accession number: PRJEB47085). Newly reported present-day mtDNA sequences are deposited into GenBank (MZ920249 - MZ921390). Additional requests should be addressed to: marina.silva@crick.ac.uk; gonzalo.oteo-garcia@hud.ac.uk; m.b.richards@hud.ac.uk.
