# Supplementary material for: Biomolecular insights into North African-related ancestry, mobility and diet in eleventh-century Al-Andalus
Source: Sci Rep. 2021 Sep 13;11:18121. doi: 10.1038/s41598-021-95996-3 (PMC8438022; doi:10.1038/s41598-021-95996-3)
Supplement: Supplementary file 1 — Supplementary Information. [file 41598_2021_95996_MOESM1_ESM.pdf]

Supplementary Information

Biomolecular insights into North African-related ancestry, mobility and diet in eleventh-century Al-Andalus

Marina Silva[1,*,+], Gonzalo Oteo-García[1,*], Rui Martiniano[2,3], João Guimarães[4], Matthew von Tersch[5], Ali Madour[1], Tarek Shoeib[1,6], Alessandro Fichera[1], Pierre Justeau[1], M. George B. Foody[1], Krista McGrath[5,7], Amparo Barrachina[8], Vicente Palomar[9], Katharina Dulias[1,5,10], Bobby Yau[1], Francesca Gandini[1], Douglas J. Clarke[1], Alexandra Rosa[11,12], António Brehm[12], Antònia Flaquer[13], Teresa Rito[4,14,15], Anna Olivieri[16], Alessandro Achilli[16], Antonio Torroni[16], Alberto Gómez-Carballa[17,18], Antonio Salas[17,18,19], Jaroslaw Bryk[1], Peter W. Ditchfield[20], Michelle Alexander[5], Maria Pala[1], Pedro A. Soares[4,21], Ceiridwen J. Edwards[1], and Martin B. Richards[1]

[1]Department of Biological and Geographical Sciences, School of Applied Sciences, University of Huddersfield, Queensgate, Huddersfield, HD1 3DH, United Kingdom;
[2]Department of Genetics, University of Cambridge, Downing Street, Cambridge CB2 3EH, United Kingdom;
[3]School of Biological and Environmental Sciences, Liverpool John Moores University, Liverpool L3 3AF, UK;
[4]CBMA (Centre of Molecular and Environmental Biology), Department of Biology, University of Minho, Campus de Gualtar, 4710-057 Braga, Portugal;
[5]BioArCh, Department of Archaeology, University of York, United Kingdom;
[6]Department of Forensic Science, Faculty of Biomedical Science, University of Benghazi, P.O. Box: 1308, Libya
[7]Department of Prehistory and Institute of Environmental Science and Technology (ICTA),Universitat Autònoma de Barcelona, 08193 Bellaterra, Spain.
[8]Servei d'Investigacions Arqueològiques i Prehistòriques - Museu Belles Arts de Castelló, Av. Germans Bou, 28 - 12003 Castellón, Spain;
[9]Museo Municipal de Arqueología y Etnología de Segorbe. Calle Colón, 98, 12400 Segorbe, Castellón, Spain;
[10]Institut für Geosysteme und Bioindikation, Technische Universität Braunschweig, Langer Kamp 19c 38106, Braunschweig, Germany;
[11]Faculty of Life Sciences, University of Madeira, Campus of Penteada, 9000-390 Funchal;
[12]Human Genetics Laboratory, University of Madeira, Campus of Penteada, 9000-390 Funchal, Portugal;
[13]Institute for Medical Information Processing, Biometry and Epidemiology - IBE, LMU University, Munich, Germany.
[14]Life and Health Sciences Research Institute (ICVS), School of Medicine, University of Minho, 4710-057 Braga, Portugal;
[15]ICVS/3B's, PT Government Associate Laboratory, 4710-057 Braga/4806-909 Guimarães, Portugal;
[16]Dipartimento di Biologia e Biotecnologie "L. Spallanzani" Università di Pavia, 27100, Pavia, Italy;
[17]Grupo de Investigacion en Genetica, Vacunas, Infecciones y Pediatria (GENVIP), Hospital Clínico Universitario and Universidade de Santiago de Compostela, Galicia, Spain
[18]GenPoB Research Group, Instituto de Investigación Sanitaria (IDIS), Hospital Clínico Universitario de Santiago (SERGAS), 15706, Galicia, Spain
[19]Unidade de Xenética, Instituto de Ciencias Forenses (INCIFOR), Facultade de Medicina, Universidade de Santiago de Compostela, Galicia, Spain
[20]School of Archaeology, University of Oxford, 1 South Parks Road, Oxford OX1 3TG, United Kingdom;
[21]Institute of Science and Innovation for Bio-Sustainability (IB-S), University of Minho, Campus de Gualtar, 4710-057 Braga, Portugal.

*These authors contributed equally to this work.

+current address: Ancient Genomics Laboratory, The Francis Crick Institute, London, United Kingdom.

Supplementary Information

Supplementary Methods. Detailed Materials and Methods

Supplementary Note 1. Phylogeography of mtDNA haplogroup U6

Supplementary Note 2. Diet and mobility in Islamic Segorbe

Supplementary Figures

Supplementary Methods

Detailed Materials and Methods

Archaeological background

The site of Plaza del Almudín in Segorbe (province of Castellón, Spain) is a medieval Islamic necropolis dated to the 11th–13th centuries CE[1] (Supplementary Figure S2). All individuals were buried according to Islamic tradition, lying on their right side, orientated northeast–southwest, and facing southeast, towards Mecca.

We were able to sample three individuals for ancient DNA analysis, but only one individual, UE2298/MS060 (the "Segorbe Giant"), excavated in 1999 (Supplementary Figure S2), yielded DNA for genomic analysis. This was a ~25-year-old male, whose burial stood out from the others in the cemetery in several ways. He was 184–190 cm tall, ~20-25 cm taller than any other individual buried at the same site, which led to his designation as "Giant". His grave was the deepest found in the cemetery, and was covered by a layer of rocks that protected the grave and contributed to the particularly good anatomical preservation of his remains[1]. This allowed for a detailed anthropological study, which concluded that he suffered from various non-lethal pathologies, impoverished nutrition and/or high-fever episodes during childhood[2]. Nevertheless, these episodes of malnutrition and/or disease did not seem to handicap his well-above-average growth. There is also evidence of porotic hyperostosis in his skeleton, most likely a result of some sort of anaemia[2].

In order to investigate his diet and mobility patterns, we also collected tooth samples from twelve additional individuals from the necropolis for dietary stable isotopic analysis (Supplementary Table S1). Although the necropolis is dated to the 11th–13th centuries CE, all human samples collected for this study came from a context dated to the 11th century CE. Additionally, we collected seventeen bone fragments from animals found in the site (although one was later identified as human by ZooMS and therefore excluded from the dataset). The faunal assemblage from Segorbe might post-date the timeframe of the Islamic necropolis of Plaza de Almudín, possibly dating instead to the later Christian period. All samples were stored in the Museo Municipal de Arqueología y Etnología de Segorbe, from where they were selected. Permissions for sample collection and analysis were agreed by the museum, and granted by the Direcció General de Cultura i Patrimoni (Conselleria d'Educació, Investigació, Cultura i Esport de la Generalitat Valenciana).

Sample processing, aDNA extraction and sequencing

We processed all the archaeological samples in clean rooms in the specialized Ancient DNA Facility at the University of Huddersfield. Surfaces and tools were frequently bleached, cleaned with LookOut® DNA Erase (SIGMA Life Sciences) and exposed to UV-radiation. We used full-body suits, gloves, hairnets and face masks at all stages. We subjected samples to UV-radiation for a total of 60 minutes (30 minutes each side), and cleaned the sampling surfaces with air-abrasion using 5µm aluminium oxide powder and a SWAM-Blaster compressed air abrasive system. We used a hobby drill with a diamond-tipped circular saw to sample the roots of the teeth, which were powdered using a Mixer Mill (Retsch MM400; 45 seconds at a frequency of 30 Hz/s), aiming for ~0.15g of fine tooth powder.

We performed DNA extraction following the protocol of Yang et al.[3] with modifications by MacHugh et al.[4], including four blanks (air, water and two extraction buffer controls) at different stages to control for contamination. We confirmed DNA extraction by DNA quantification with Qubit™ 3.0 Fluorometer (ThermoFisher Scientific), using the Qubit® dsDNA HS Assay Kit.

We performed library preparation following the protocol by Meyer and Kircher (2010), with modifications as described in Gamba et al.[6] and Cassidy et al.[7]. Initially we sequenced one USER™-treated library on one tenth of an Illumina HiSeq4000 lane (100 cycles) to screen for endogenous aDNA content. We then ran three additional libraries (one of which was non-USER treated) on half an Illumina HiSeq4000 lane, sequenced for 100 cycles. Sequencing was performed at Macrogen, inc. (Seoul, South Korea).

Sequence data processing

We assessed raw read quality by processing *fastq* files with FastQC v.0.11[8], and merged paired-end reads and removed sequencing adapters using leeHom[9]. We mapped reads both to the human genome reference (hg19, but modified to include rCRS instead of chrM) and to only the rCRS (revised Cambridge Reference Sequence, GenBank accession code: NC_012920) with BWA (Burrows-Wheeler Aligner) v.0.7.5a-r405 *aln*[10] (using the optimized settings for aDNA mapping: -l 16569, -n 0.01 and -o 2[11]) and *samse*. We used samtools v.1.4[12] to sort *bam* files (*sort*), remove PCR duplicates (*rmdup*) and to exclude reads below mapping quality 30 and minimum read length of 30 base pairs (bp).

We performed quality control of the alignment with QualiMap v.2.2.1[13]. To confirm aDNA authenticity, we (1) checked for contamination (0–0.5%) on the mtDNA sequence of the non-treated library using schmutzi[14]; (2) confirmed one single mtDNA haplotype across all libraries, consistent with one single donor (Supplementary Table S2); (3) confirmed that all libraries could confidently be assigned as male[15] (Supplementary Figure S3); (4) estimated contamination in the X chromosome using ANGSD[16] (Method 1, new_llh (MoM): 0.037142±3.745333e-2); and (5)

confirmed post-mortem damage and DNA fragmentation with both mapDamage v. 2.0.7[17] and bamdamage (included in bammds package[18]) (Supplementary Figure S12). USER-treated libraries displayed a lower percentage of post-mortem damage, usually restricted to only the terminal nucleotide, whereas the non-treated library, as expected, presented a higher percentage of damage (Supplementary Figure S12).

We followed two approaches to avoid the effect of SNP miscalls due to post-mortem misincorporations: (1) we downscaled base quality scores of positions likely affected by post-mortem damage using the *--rescale* option in mapDamage, and (2) we soft-clipped the terminal 3 bp of reads with the *trimBam* option in bamUtil v. 1.0.14[19], to control for potential reference bias resulting from downscaling base quality scores that could influence formal tests of admixture and *qpAdm* modelling. We then merged all libraries using picard *MERGESAM* (https://github.com/broadinstitute/picard). Published ancient samples included in the autosomal analysis were remapped and reanalysed alongside UE2298/MS060 to prevent possible batch effects due to differences in pipelines.

Analysis of mtDNA and Y-chromosome variation

We retrieved mtDNA variant positions (Supplementary Table S2) with GATK v.3.7-0-gcfedb67 HaplotypeCaller[20] and performed haplogroup assignment using HaploGrep 2.0[21], according to the nomenclature of PhyloTree (Build 17, February 2016)[22]. We checked individually all missing and private mutations detected by HaploGrep, as well as heteroplasmies, with IGV v.2.3[23].

We classified Y-chromosome variation into haplogroups using Yleaf[24], and checked mutations against the ISOGG (International Society of Genetic Genealogy) SNP index (as of June 2018) (Supplementary Table S2). Next, we used pathPhynder[25] to investigate the affinity of our medieval sample with present-day Y chromosomes. This approach is ideal for low-coverage ancient samples such as UE2298/MS060, which has only ~9000 reads aligning to the Y chromosome, because it uses all available variation within a present-day-dataset, including SNPs not yet catalogued in ISOGG, and therefore potentially increasing the number of SNPs overlapping with an ancient individual[25]. For this analysis, we merged a subset of 256 individuals from the Hallast et al.[26] and Solé-Morata et al.[27] datasets and estimated a neighbour-joining tree with the ‘ape’[28] R[29] package. We used the pathPhynder software to assign informative SNPs to tree branches, which we subsequently called in the ancient sample, using them to infer the individual’s most likely position within the phylogeny.

Autosomal analysis

We called a total of 74,209 pseudo-haploid autosomal SNPs (72,280 when soft-clipping the terminal 3 bp of reads) against the 1240k SNP list (available at https://reich.hms.harvard.edu/)

using samtools *mpileup* (-R, -B, -q30, -Q30) and *pileupCaller* (https://github.com/stschiff/sequenceTools). Principal component analysis (PCA) of autosomal SNPs was performed on a subset of ~600k autosomal SNPs using smartpca (part of EIGENSOFT package[30]), with the default setting for outlier removal, *shrinkmode: YES* and *lsqproject: YES* to project 336 ancient samples on a selection of 702 modern individuals from North Africa, Europe, the Caucasus and the Near East[31] (Supplementary Table S3).

We compiled a dataset with ~1.2M SNPs for analysis including only ancient samples. We filtered this for positions in linkage disequilibrium (LD) using the command *--indep-pairwise* (200, 25, 0.4) in PLINK v.1.07[32], resulting in 451,051 SNPs. As an exploratory analysis, and following evidence presented by Olalde et al.[33] for North African and Levantine-related ancestry in Iberia from at least the third century CE, we ran ADMIXTURE v.1.3.0[34] for post-Iron Age Iberian individuals (with parameters: *--cv* and *--seed time*) in supervised mode for K=3, using *Iberia_IA*, *Morocco_LN/Guanches* and *Levant_BA* as reference populations.

The following tests were performed using two datasets generated using different strategies to deal with post-mortem damage ("mapDamage *--rescale*" and "soft-clipping"). We ran outgroup-*f3* statistics using *qp3Pop*, with three different outgroups (*Mbuti*, *Ju_hoan_North*, *Ust_Ishim*)[35,] to account for potential divergent human ancestry (North African and European) in the genome of UE2298/MS060. We also ran *D*-statistics (using a chimpanzee genotype and Mbuti as outgroups) with *qpDstat* (both included in ADMIXTOOLS v.4.1[36]) to untangle Iberian and North African-related contributions, as follows: *D(outgroup, UE2298/MS060; Iberian population, North African population)*. *D*-statistics was also applied with the formula *D(outgroup, UE2298/MS060; Morocco_LN, Guanches)* for more refined insights into the ancestry of individual UE2298/MS060.

We used *qpAdm*[37] (ADMIXTOOLS v.4.1) testing 1- and 2-way models, using different combinations of *Morocco_LN/Guanches* and ancient Iberian groups as source (left) populations, with *allsnps: YES*, and using a set of outgroups (right populations) based on that used by Olalde et al.[33] for southeast Spain in the last two millennia: *Belgium_GoyetQ116_1, Czech_Vestonice16, EHG, Ethiopia_4500BP.SG, CHG, Iberia_ElMiron, Russia_Kostenki14, Russia_MA1_HG.SG, Ust_Ishim.DG, Yamnaya, LBK_EN, Anatolia_N, Natufian, Iberia_Meso, Poland_Globular_Amphora, Morocco_EN, Iberomaurusian_Morocco.*

All plots were computed either using basic R plot options[29], or *ggplot2* package[38] in RStudio[39].

Modern Iberian mtDNA dataset

We newly sequenced complete mitogenomes of a subset of 1023 samples from the Spanish National DNA Bank project (http://www.bancoadn.org), as previously described in Barral-Arca et al.[40] (1000 assigned to mainland Spain, 8 to the Balearic Islands, 11 to the Canary Islands and

4 to Melilla and Ceuta), and 103 samples from present-day Portugal (96 from mainland and 7 from Madeira archipelago) (GenBank accession codes: xxxxxx-xxxxxx). Geographic origins were assigned based on the birthplace of the donors' maternal grandmother. For Portuguese samples, we performed DNA extraction using a salting-out protocol (adapted from Miller et al.[41]) (in the case of PT-codes) or with Quick-DNA™ Universal Kit (Zymo Research) (in the case of POR-codes). We amplified all samples using long-range PCR with conditions optimized according to the specifications of the GoTaq® Long PCR Master Mix kit (Promega Corporation) (primer sequences in Supplementary Table S13). PCRs contained 0.5µL of template DNA, 11µL of nuclease-free water, 1xGoTaq® Long PCR Master Mix (Promega), and 0.2 µM of each primer (final volume 25µL). The PCR program consisted of an initial denaturation step of 2 minutes at 94°C, followed by 30 cycles (denaturation at 94°C for 30 seconds, primer annealing at 55°C for 30 seconds and extension for 9 minutes at 65°C) and final step of 10 minutes at 72°C[42]. We confirmed amplification with 1% agarose gel electrophoresis. We performed column purification of PCR products according to Wizard® SV Gel and PCR Clean-Up System (Promega) protocol and quantified the purified DNA with Qubit™ 3.0 Fluorometer (Qubit® dsDNA HS Assay Kit). We then pooled and sent samples for library preparation (using an optimized protocol based on Nextera® DNA Library preparation kit (Illumina, Inc)) and sequencing with Illumina MiSeq paired-end sequencing (size of fragment: 150 bp) at the Earlham Institute, located at the Norwich Science Park (United Kingdom).

We used the EAGER (efficient ancient genome reconstruction) pipeline[43] for many initial steps. We checked raw FASTQ files with FastQC[8] and ran through AdapterRemoval v.2.2[44] to remove adaptors and merge paired reads. We aligned the reads to rCRS with BWA-MEM[10], which is optimized for long Illumina reads. We identified PCR duplicates with DeDup (included in EAGER pipeline) and performed quality control of the alignment with QualiMap v.2.2.1[13]. We ran GATK v.3.7-0-gcfedb67[20] Indel Realigner and HaplotypeCaller. We performed the initial SNP filtering for minimum quality (Q30) and minimum coverage (5x) using VCFtools v.0.1.11[45] and then handled the resultant VCF file with BCFtools v.1.4 *view* and a shell command in order to further filter the called variants: we scored a polymorphism when minimum allele frequency was over 0.70 (MIN(AF)>0.7), discarding SNPs with frequency under 0.30, and we called all remaining variants to a separate file as potential heteroplasmic positions, which we then manually checked with IGV.

We assigned samples to haplogroups using HaploGrep 2.0[21], using nomenclature from PhyloTree (build 17, February 2016)[22]. We computed density distribution maps for mtDNA haplogroups H (H1, H2, H3, H4, H5), HV, J, I, K, L, M1, T, U(xU6), U6, U6a, V, and X in present-day Iberia using a subset of this dataset (*n*=1104, excluding sequences assigned to the Canary Islands, Melilla and Ceuta, and Madeira archipelago (Supplementary Table S5), using a 2D kernel density estimator in R[29,39] with the occurrence of each haplogroup. Coordinates of

Spanish provincial capital cities were used as calibration points, whereas for the Portuguese dataset we considered three geographic points (Porto, Aveiro and Lisbon).

Phylogeographic analysis of mtDNA haplogroup U6

We built a phylogenetic tree of mtDNA haplogroup U6 based on a total of 330 modern (35 of which are newly published here) and 32 ancient sequences (one being UE2298/MS060) using MtPhyl v.5.003 software (http://eltsov.org) (Supplementary Table S4). The newly reported present-day mitogenomes comprise 19 sequences from Portugal and Spain (included in the dataset described above), 9 from Libyan Berbers (DNA extracted from buccal swabs with QIAamp® DNA blood mini kit, by QIAGEN®), 3 from Italy (DNA extracted from saliva following a standard phenol/chloroform protocol), and 4 sampled in Germany for the KORA study[46]. We amplified and sequenced samples from Libya and Italy using the protocols described above for the Iberian dataset; sequences from Germany were generated as part of the KORA study. We assigned geographic origins of newly reported present-day samples based on the birthplace of the donors' maternal grandmother.

We excluded insertions at positions 309 and 315, indels between positions 515 and 522, and hotspot positions 16182, 16183 and 16519 from the tree, in accordance with PhyloTree recommendations. Node age estimates were calculated from present-day sequences using rho (ρ) statistic and maximum likelihood (ML). We excluded remaining indels (included in the phylogenetic reconstruction), as they are not considered by the models used for age calculations. We used a mutation rate of one substitution in every 3,624 years, correcting for purifying selection[47], estimating standard errors as in Saillard et al.[48]. We performed ML estimates of branch lengths using PAML 4[49], assuming the HKY85 mutation model with gamma-distributed rates (discrete distribution of 32 categories) and considering two partitions so as to differentiate the fast-evolving hypervariable segments (HVS) I (16024-16400 bp) and II (44-340 bp) from the rest of the molecule.

We computed Bayesian skyline plots (BSPs)[50] for the complete modern U6 dataset using BEAST v.1.8.0[51] (100,000,000 interactions with a burn-in of 10,000,000 steps), applying a relaxed molecular clock with a mutation rate of 2.514×10^{-8} mutations/site/year (previously calculated for U6[52]), assuming a 28-year generation time[53]. We combined three independent runs with LogCombiner v.1.8.0, included in BEAST package.

Mobility isotope analysis: oxygen

We selected samples for enamel carbonate analysis based on tooth type (Supplementary Table S1), avoiding teeth that would be affected by nursing enrichment where possible[54]. We avoided the top ~2mm of the tip of the crown where enamel forms first when sampling second molars and premolars to do our best to ensure that the majority of the bulk sample was made up

of enamel formed after nursing had probably ceased, although with possible 1st/2nd molars and 1st/2nd premolars some nursing effect may be present if these are indeed the earlier forming teeth. Historical sources indicate that medieval populations were typically breastfed to at least two years of age[55].

For enamel carbonate sampling and pre-treatment, we followed that described by Ventresca Miller et al.[56]. We cleaned the outer surface of each tooth crown using an aluminium oxide shot-blaster. We then took tooth enamel powder samples in bulk across the whole surface of the tooth from just above the cementoenamel junction to almost the top of the occlusal surface (leaving ~2mm from the top) using a hand-held drill with a diamond-tipped burr. Pre-treatment to remove exogenous carbonates was carried out by adding 0.1ml of 0.1M of acetic acid per mg of enamel to each sample, which was left for 10min. The acetic acid was then removed and the samples rinsed three times before being freeze-dried. Enamel apatite $\delta^{13}C$ and $\delta^{18}O$ were analysed at Iso-Analytial, Cheshire, UK, by Continuous Flow-Isotope Ratio Mass Spectrometry (CF-IRMS) using a Europa Scientific 20-20 mass spectrometer. Powdered samples and controlled carbonates were weighed into Exetainer™ tubes (Labco, UK) which were then flushed with 99.995% helium, and the samples converted to carbon dioxide by injecting phosphoric acid. Control material analysed included IA-R022 (Iso-Analytical working standard calcium carbonate, $\delta^{13}C$ V-PDB = –28.63‰ and $\delta^{18}O$ V-PDB = –22.69‰), NBS-18 (carbonatite, $\delta^{13}C$ V-PDB = –5.01 ‰ and $\delta^{18}O$V-PDB = –23.20 ‰) and IA-R066 (chalk, $\delta^{13}C$ V-PDB = +2.33 ‰ and $\delta^{18}O$ V-PDB = –1.52 ‰). Standard deviations of repeated measurements of these standards were ≤0.2‰ for both $\delta^{13}C$ and $\delta^{18}O$. Carbonate $\delta^{18}O_{VPDB}$ values are reported as $\delta^{18}O_{VSMOW}$.

Dietary isotope analysis

We selected teeth from a total of 13 individuals from the necropolis, including UE2298/MS060. These included two incisors, two premolars, one canine and eight molars (Supplementary Table S1). Due to the fragmentary nature of the animal remains collected at the necropolis of Plaza de Almudín (Segorbe), bone samples were identified to species level by ZooMS (Zooarchaeology by Mass Spectrometry) (Supplementary Table S12). Carbon ($\delta^{13}C$) and nitrogen ($\delta^{15}N$) stable isotope analyses were undertaken at the Research Laboratory for Archaeology at the University of Oxford. Collagen from the tooth root was prepared and demineralised using 10ml aliquots of 0.5M HCl solution at 4°C. The acid solution was changed at 48-hour intervals until no further reaction was seen. The sample was then rinsed three times with milli-μ ultra-pure water and placed in 10ml of pH 3 water at 75°C for 48 hours. The resulting solution was filtered using an Eezee™ filter, and the supernatant liquid decanted into a Nalgene™ tube with a temporary Parafilm™ cover. We pre-froze the sample at -40°C prior to freeze drying in a Zirbus VaCo5 freeze drier fitted with an oil-free vacuum system for 72 hours.

The resulting purified collagen was weighted out for analysis using ~1mg aliquots weighed into pre-cleaned tin capsules. Samples were combusted on a Sercon GSL elemental analyser system using a helium carrier gas with a flow of approximately 80ml per minute. A 2% split of the gases evolved for nitrogen and carbon were analysed for stable isotopic composition using a Sercon -20/22 gas source mass spectrometer. Isotopic values as well as elemental abundances and carbon-to-nitrogen ratios were calibrated against an Oxford *in-house* alanine standard, which itself is routinely measured against international standards (USGS 40 and USGS 41 glutamic acid, whose values are traceable back to the V-PDB and AIR international standards for carbon and nitrogen, respectively). Further aliquots of the alanine standard were used to monitor and correct for instrumental drift. The samples were run in duplicate. The stable isotopic results are in delta notation relative to V-PDB for carbon and AIR for nitrogen. Replicate analysis of the alanine in-house standard gave the following results (n = 10 measurements): Mean $\delta^{13}C$ = –27.15 ± 0.13‰, expected value –27.11‰; mean $\delta^{15}N$ = –1.55 ± 0.15‰, expected value –1.56‰. This suggests that the individual values obtained during the analyses reported here are typically accurate to better than ± 0.1‰ for both $\delta^{13}C$ and $\delta^{15}N$. The faunal base line incorporated data is both from this study and the nearby site of Benipeixcar (Gandía, Valencia)[57]. We compared our human results with published data from other medieval archaeological sites from eastern Spain, Gandía and Valencia[57,58].

FRUITS model

The proportional contribution of different food sources to the human diet at Segorbe was explored using the FRUITS v.3 Bayesian mixing model[59], inputting the stable isotope values for both humans and their potential dietary sources. Three dietary proxies were used, $\delta^{13}C_{col}$, $\delta^{15}N_{col}$ (bone) and $\delta^{13}C_{ap}$ (tooth enamel). As BMMs decrease in power with increasing number of sources[60] and there is no evidence for C_4-fed animals at Segorbe itself, four source food groups were used for the purposes for the FRUITS model: C_3 plants, C_4 plants, terrestrial herbivores and marine fish. The mean values for terrestrial animal $\delta^{13}C_{col}$, $\delta^{15}N_{col}$ and $\delta^{13}C_{ap}$ (tooth enamel) were derived from the present study. Marine fish data derived from published data from medieval sites in Spain (mean $\delta^{13}C_{col}$ = -10.8 ± 0.8‰ $\delta^{15}N_{col}$= 10.8 ± 1.9‰, n=5)[57]. There is no published plant isotope data from medieval Iberia, so the mean isotopic values for both C_3 (wheat and barley, mean $\delta^{13}C$= -22.2 ± 0.5‰ $\delta^{15}N$= 7.0 ± 0.8‰, n=21) and C_4 (millet, *Panicum milliaceum,* mean $\delta^{13}C$= -10.8 ± 0.2‰ $\delta^{15}N$= 10.2 ± 0.4‰, n=3) plants were derived from La Hoya, an Iron Age site in Northern Iberia where a variation in manuring and crop water management took place[61]. The plant data had already been corrected for charring by the original authors[61].

We used a concentration dependent and routed model for the Bayesian estimations. Lipids and carbohydrates were combined into a single 'energy' fraction. The macronutrient composition (protein, carbs/lipids) of food groups was estimated following Fernandes et al.[62]. The average

$\delta^{13}C$ and $\delta^{15}N$ values of the nutrient fraction (protein, carbohydrate/lipids) of each food source was derived from bone collagen values and estimated using fractionations reported in Fernandes et al.[63]. These were: $\Delta^{13}C_{protein-collagen}$ =-2 ‰, $\Delta^{13}C_{lipids-collagen}$ =-8‰ and $\Delta^{15}N_{protein-collagen}$=+2‰ for terrestrial mammas and $\Delta^{13}C_{protein-collagen}$ =-1 ‰, $\Delta^{13}C_{lipids-collagen}$ =-7‰ and $\Delta^{15}N_{protein-collagen}$=+2‰ for fish. The offset for plants assumed the $\delta^{15}N$ value of plant protein was the same as the average bulk plant $\delta^{15}N$ value and for $\delta^{13}C$, the offsets were $\Delta^{13}C_{bulk-protein}$= -2 ‰ and $\Delta^{13}C_{bulk-carbohydrates}$ = +0.5 ‰. A conservative uncertainty of 1‰ was used for all offsets. $\delta^{13}C_{ap}$ values represent the total carbon in the diet and so the weighted average of each food group according to nutrient composition was considered as the source of the $\delta^{13}C_{ap}$ values[62]. The model assumed nitrogen isotopes were sourced solely from proteins (100%), whereas to account for carbon routing, proteins were assumed to account for 74 ± 4% of collagen carbon, with lipids and carbohydrates (energy) contributing the remaining 26.5%[63]. Diet-tissue offsets between diet-to-collagen and diet-to-apatite $\delta^{13}C$ (+5 ± 0.5 ‰ and +10.1 ± 0.5 ‰, respectively), and diet-to-collagen $\delta^{15}N$ (+5.5 ± 0.5 ‰) were derived from Fernandes et al.[63]. The estimations for the diet were constrained to protein contributing >5% and <45% of the total calorific intake[59].

Identification of animal bone by collagen peptide mass fingerprinting (ZooMS)

ZooMS is a qualitative analytical technique for taxonomic identification of archaeological bone through collagen peptide mass fingerprinting[64,65]. Analysis was undertaken on a sub-sample of the extracted collagen from all seventeen animal samples. Approximately 0.5mg of extracted collagen was added to 50µL of AmBic (ammonium bicarbonate buffer, pH 8.0) and vortexed. 0.4µg of trypsin (Promega) was added and the samples were digested overnight at 37°C. Afterwards, samples were centrifuged for 1 minute at 13,000rpm, following which 1µl of 5% TFA was added to stop enzymatic digestion. A C18 ZipTip (Agilent) was used for peptide extraction, and eluted using 50µl of 50% ACN in 0.5% TFA.

MALDI-TOF-MS analysis followed previously established protocols[64], but using 1µl sample solution and 1µl matrix (α-cyano-hydroxycinnamic acid) solution. These were spotted in triplicate along with calibration standards onto a Bruker ground steel target plate and run on a Bruker ultraflex III MALDI TOF/TOF mass spectrometer. Spectra were analysed using the mMass software[66]. We assigned taxonomic identifications based on the presence of unambiguous m/z markers (Supplementary Table S12) from published sources[64,67,68]. One bone fragment, GOGa03, was determined to be a human and was not included in the faunal isotopic study.

Supplementary Note 1

Phylogeography of mtDNA haplogroup U6

The study of individual UE2298/MS060 prompted a renewed phylogeographic study of mtDNA haplogroup U6 (Supplementary Figure S4). Haplogroup U6 dates to ~39.8 [29.2–50.7] ka, based on our maximum likelihood (ML) analysis. Its modern distribution differs sharply from other haplogroup U subclades, which are mostly west Eurasian lineages, with the typically European U4 and U5 representing the majority of Mesolithic European lineages described[69–71], U3 and U8 (including K) spanning Europe and Southwest Asia[72], U1 restricted to the Near East[73,74], and U2 and U7 spanning west Eurasia but found predominately in Southwest and South Asia[75,76].

U6, on the other hand, has a broad Mediterranean distribution, with its highest extant frequencies in North Africa, and some subclades present in sub-Saharan Africa. However, basal U6* has been retrieved from two samples dating to 35–33 ka from the Peştera Muierii cave in Romania[77,78] (recently confirmed to belong to the same individual[79]; labelled here as PM1/Muierii2) and an additional Palaeolithic U6 lineage from the Caucasus (~27–24 ka) was recently described[80], suggesting that U6 most likely originated in Eurasia, in common with other haplogroup U subclades, and was later involved in a pre-Holocene back-to-Africa migration, probably from Southwest Asia[81–84]. This must have occurred before 14–15 ka (the age of the Iberomaurusian remains in Taforalt, Morocco, the earliest known U6 lineages in North Africa[85]). The expansion of the Iberomaurusian culture into Northwest Africa dates to, or precedes, the early Last Glacial Maximum (LGM) (at least 25 ka)[86].

U6a is the largest and most widespread of all U6 clades, and the only one estimated to pre-date the LGM (26.9 [21.4-32.4] ka). U6a seems likely to have been restricted to the Mediterranean basin before the LGM, where its oldest branches arose: U6a1 (21.7 [14.5-29.2] ka) and U6a7 (25.2 [19.4-31.2] ka) both harbour the bulk of Iberomaurusian and Early Neolithic (EN) Moroccan lineages[85,87]. In contrast, sub-Saharan branches (either in west or east Africa) all date to the post-LGM. U6a3 (18.7 [14.3-23.3] ka) shows a dual distribution, with lineages spanning from the east (U6a3d: 7.3 [1.2-13.5] ka) to the west Mediterranean (U6a3a: 12.9 [6.7-19.4] ka), and sub-Saharan west African lineages with ages ranging from the Late Glacial and early postglacial to the Neolithic: U6a3f (15.4 [9.7-21.2] ka), U6a3+150 (9.8 [2.8-17.1] ka) and pre-U6a3c (5.0 [0.7-9.5] ka). U6a2a (12.3 [7.0-17.8] ka) and U6a2b (9.2 [1.9-16.9] ka) date to Late Glacial/postglacial Ethiopia. U6a8, harbouring North African and southern European sequences, also dates to post-LGM/Late Glacial period (14.9 [6.5-23.8] ka).

Haplogroup U6a1, dating to 21.7 ka, has been found both in Iberomaurusian samples dating to 14–15 ka[85] and in Early Neolithic Morocco[87] (Figure 1b). Many of the Iberomaurusian lineages

cluster with Early Neolithic or present-day Moroccan lineages, showing a certain degree of continuity in the Maghreb on the female line of descent, despite more recent population events, such as the Arab Conquest and increase in sub-Saharan influx, that shifted the autosomal variation in the region[88]. To our knowledge, UE2298/MS060 is the earliest documented finding of a U6 lineage in mainland Iberia. Today, U6a is found at very low frequencies in the Iberian Peninsula. In our large dataset of complete modern mtDNA sequences (*n*=1104 from mainland Spain, the Balearic Islands, and mainland Portugal), U6a has an average frequency of 1.6% in the peninsula, with a peak of 3.57% in the south of Spain (Figure 1b). However, the city of Segorbe falls outside the modern distribution of U6 lineages in Spain, suggesting that U6 could have been more widespread in medieval times.

U6b (12.5 [8.6-16.5] ka), U6c (10.8 [5.4-16.3] ka) and U6d (12.7 [7.4-18.2] ka) are smaller subclades dating to the Pleistocene–Holocene transition. U6c is present in the western Mediterranean region and in the Canary Islands, whereas U6b and U6d have a wider distribution around the Mediterranean basin, with incursions into sub-Saharan Africa, North Europe and Arabia. U6b1a is restricted to the Canary Islands, including in remains from Guanches, Canary Island aboriginals from the 7th–11th centuries CE, who settled from Morocco ~2.5 ka[89,90]. Its age estimate of 2.8 [0.9-4.6] ka suggests that this lineage was likely carried by the first settlers of the islands[91].

The Bayesian skyline plot (BSP) of haplogroup U6 indicates three main episodes of population increase (Supplementary Figure S13): (1) around the LGM, just before 20 ka, most likely a trace of Iberomaurusian expansions in North Africa, as mentioned above and discussed further in Pereira et al.[52]; (2) in the Late Glacial (12–15 ka), coinciding with the ranges for the age estimates of the U6 branches in sub-Saharan Africa in our tree; and (3) in the last 5 ka, with an acceleration in the last ~2 ka.

The main feature of the glacial period in Africa was high aridity, with the expansion of the Sahara hundreds of kilometres southwards[92]. This probably prevented contacts between north African and sub-Saharan populations and explains the scarcity of evidence for human occupation during this period. During the African Humid Period (~14.7–5.5 ka), the north and central African climate was more moist than today[93,94]. The expansion of rainforest in equatorial Africa, combined with the movement of monsoon rains northwards, resulted in the greening and occupation of the Sahara[92,95–97], probably potentiating the population increase associated with U6 observed in the BSP and movements into Eastern and West Africa, with the rise of several U6 sub-Saharan clades (e.g.: U6a2a, U6a2b, U6a3c and U6a3f). The 5-ka increment might correspond to the expansion of indigenous communities triggered by the spread of agriculture into the Maghreb[87], and the more recent acceleration to further expansions set in motion by the Arab conquest of north Africa and Iberia, which led to profound cultural, religious and linguistic changes.

Supplementary Note 2

Mobility and diet in Islamic Segorbe

Mobility

For the purposes of comparison between UE2298/MS060 and the remainder of the population, this analysis was treated as an individual dataset to determine whether the enamel carbonate $\delta^{18}O$ possessed by UE2298/MS060 sets him apart from other individuals sampled from the same cemetery which would identify him as a potential first generation migrant[98]. Carbonate $\delta^{18}O_{V\text{-}PDB}$ values are reported as $\delta^{18}O_{VSMOW}$ following Coplen et al.[99] and further converted to drinking water (dw) using Daux et al.'s equation 6[100,101] for the purposes of interpretation, although it is acknowledged that such conversions are associated with issues in equation selection and error propagation (see Pederzani and Britton[102] for discussion). Tooth enamel carbonate data is presented in Supplementary Table S10 and plotted in Figure 5a. The $\delta^{18}O$ values for the Segorbe population (excluding outlier MS075, see below) range from 26.2‰ to 27.6‰ (range = 1.4‰, n=7), with a mean of 26.8 ± 0.5‰ (1σ). These $\delta^{18}O$ results are in keeping with a warm Mediterranean climate[98]. The $\delta^{18}O$ values for the earlier (M1/M2, 27.2‰) and later (M3, 26.4‰) forming teeth of UE2298/MS060 only differ by 0.8‰ and both fall within the narrow range (<2‰) of $\delta^{18}O$ values exhibited at the cemetery (Supplementary Table S10). This provides no indication of movement between early childhood and adolescence and if this molar is indeed an M1, a nursing effect could account for the slight difference between these teeth. The converted $\delta^{18}O_{dw}$ values for the population, including UE2298/MS060 (mean 6.2‰ ± 0.7‰, excluding MS075) also fit with the meteoric water values for the eastern Iberian coast ($\delta^{18}O_{VSMOW}$ -6.2 to -4.4 ‰ annual average 2004 to 2010, Valencia station[103]). Altogether, the oxygen data for UE2298/MS060 is consistent with someone who grew up in the region, however, it should be noted that the $\delta^{18}O_{dw}$ values exhibited by this population (excluding MS075) are also in keeping with those reported over a relatively broad geographical range including areas of the southern Mediterranean and North Africa[98], a possible area of migration. The identification of UE2298/MS060 as a local on the basis of this evidence is therefore not unequivocal and another proxy such as strontium isotope analysis could be used to provide more definition to local values

The M2 for one other individual in the population, MS075, however, possesses a $\delta^{18}O$ value of 30.6‰, which is more than 1.5 times the interquartile range above quartile 3. Even taking the small sample size (n <25) into account, this sample can be identified as an outlier[98] and therefore a potential migrant from a hotter environment. Consumption of a local but differing water source or significantly different food and drink preparation habits[104] in childhood cannot be ruled out, but the magnitude of the difference makes this scenario unlikely. When the carbonate value for

MS075 is converted to phosphate for comparison with published data (following the equation of Chenery et al.[100]), the $\delta^{18}O$ phosphate value of 21.9‰ is slightly less than that reported for Nubian mummies[105] and the $\delta^{18}O_{dw}$ value is within the range of values recently reported for archaeological individuals buried in Egypt[106] and in keeping with modern meteoric precipitation values for Africa or the Middle East[107]. The dietary isotopes indicate that MS075 consumed a similar diet to the rest of the population (see below), however a mixed C_3/C_4 diet is also a possibility for Africa[108].

Diet

There is a general problem of equifinality when analysing consumer isotope values in the Mediterranean[57,58]. The possibility of C_3 and C_4 plants both being available for human and animal consumption coupled with the availability of marine foods create potentially confounding effects when trying to understand archaeological human diet.

Segorbe is 35 km from the port city of Sagunto and well connected through the old Roman road connecting the valley. In the Middle Ages fish arrived from the Moncófar port, and there is a document by Martín el Humano granting the monks of Cartuja de Vall de Cristo the privilege of receiving the fish before the city of Segorbe (14th century CE)[109]. Therefore, it is not unreasonable to expect fish contribution to the diets of the Islamic inhabitants of Segorbe. Other contemporaneous sites in Spain, such as El Raval (in the city of Alicante)[110] and Valencia[58], also show evidence for a significant C_4 protein input into the human diet. Similarly high $\delta^{15}N$ values as seen in Segorbe may reflect aridity in the local area, as reported from Tauste in northern Spain[111]. Thus, the consumption of marine foods, C_4 or C_3-fed terrestrial animal protein, or C_4 crops (directly), or a combination of all three could all play a role in the human collagen dietary isotopic signal.

Preliminary analysis of archaeological faunal collagen from Segorbe (n=16) indicates that domestic animals were foddered on a largely C_3-based regime and that local aridity was not driving elevation in nitrogen isotopic composition (herbivore sheep, goat and cattle mean $\delta^{13}C$= -19.8‰, $\delta^{15}N$= 5.2‰, n=14)) (Supplementary Table S11). If these faunal values are representative of the terrestrial fauna available to the humans represented in the necropolis of Plaza del Almudín, it would suggest that the elevated carbon and nitrogen isotopic values seen in the human collagen are likely to reflect a significant but varying component of marine derived protein in the human diet.

The human data, however, lack a significant positive correlation between $\delta^{13}C$ and $\delta^{15}N$ (r^2=0.086, Pearson's r=0.33) usually characteristic of mixed terrestrial C_3/marine protein intake[111]. Any marine component to the diet is likely to be overemphasised in $\delta^{15}N$ values as some proportion of the carbon in collagen will reflect the consumption of carbohydrates and lipids in addition to protein (see Craig et al.[113] for discussion). A weak correlation between $\delta^{13}C$ and $\delta^{15}N$

values for collagen in similar contexts in Spain has therefore been taken to indicate some direct consumption of C_4 crops by humans in a C_3/marine protein-dominated system[114,115]. Given that humans from Plaza del Almudín possess collagen isotope values within the range of medieval populations from Galicia, who subsisted on both marine fish and C_4 crops in addition to C_3 terrestrial resources[115], it is probable that this dietary combination was also consumed by the human population at Sergobe. In addition, enamel carbonate values that reflect whole diet (lipids, carbohydrates, proteins) are higher at Sergobe ($\delta^{13}C_{ap}$ mean = -6.5‰, *n*=8) than published apatite values for medieval populations from Spain that are interpreted as consuming C_4 crops (Écija $\delta^{13}C_{ap}$ mean -11.5‰, *n*=40[116]; Eivissa $\delta^{13}C_{ap}$ mean -9.6‰, *n*=6[114]), which altogether serve to support this hypothesis.

Although he cannot be classed as an outlier, the collagen of UE2298/MS060 shows more negative $\delta^{13}C$ and lower $\delta^{15}N$ values than the majority of the humans from this assemblage and thus it is likely that the diet of this individual had a lower input of marine protein/C_4 crops compared to others among the Segorbe population (Supplementary Figure S11).

Supplementary Figures

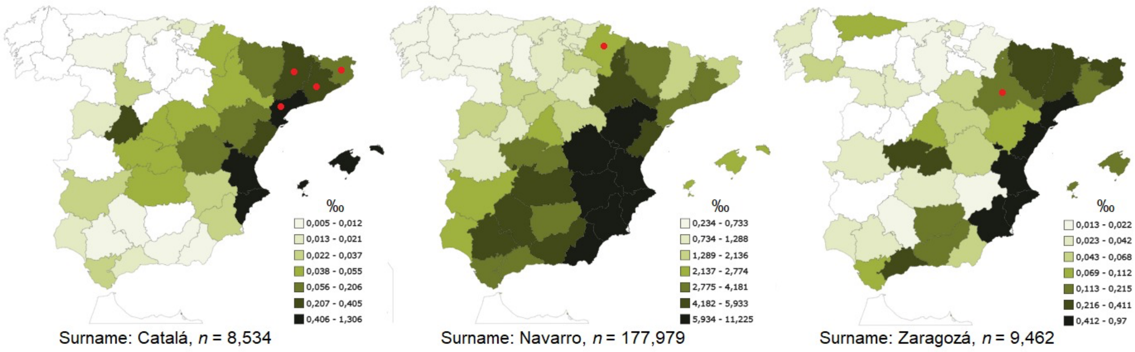

Supplementary Figure S1. Present-day frequency of surnames of new settlers in the Valencian region. Data from 2017, published by the Spanish National Institute of Statistics/Instituto Nacional de Estadística in 2018 (source: INE website, www.ine.es). Red dots represent the regions to which each surname refers: Català refers to a person from Catalonia; Navarro refers to a person from Navarre; Zaragozá refers to a person from the Aragonese city and/or Province of Zaragoza.

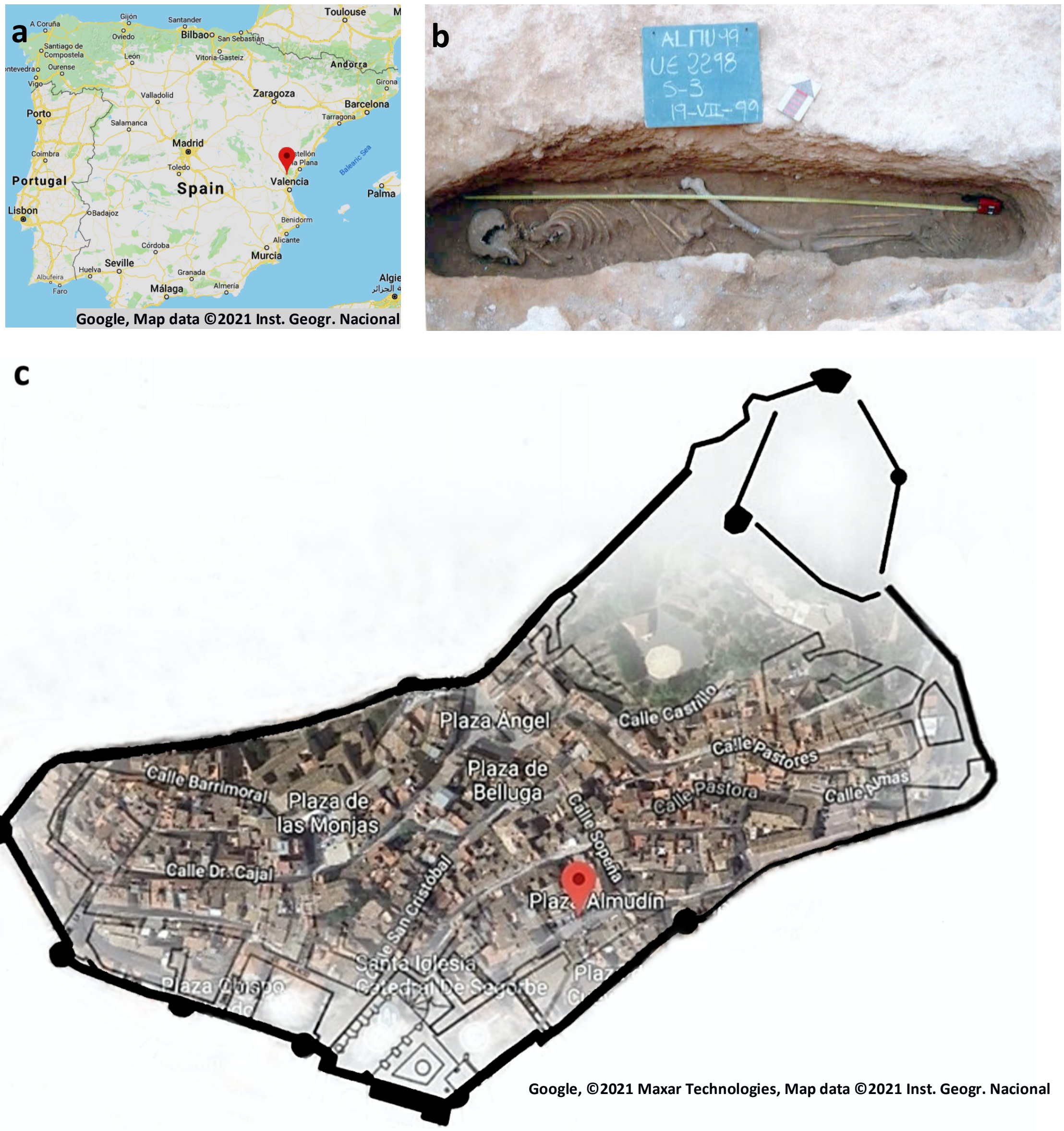

Supplementary Figure S2. a) Location of Segorbe, in the province of Castellón, autonomous community of Valencia (Spain) (image from Google Maps). **b)** Photo of individual UE2298/MS060 (known as the “Segorbe Giant”) in his burial site[1]. **c)** Plan of the Medieval (post-Islamic) city walls of Segorbe overlapped to a satellite view of modern Segorbe. Location of the archaeological site (Plaza del Almudín) indicated by the red icon. Satellite image from Google Maps.

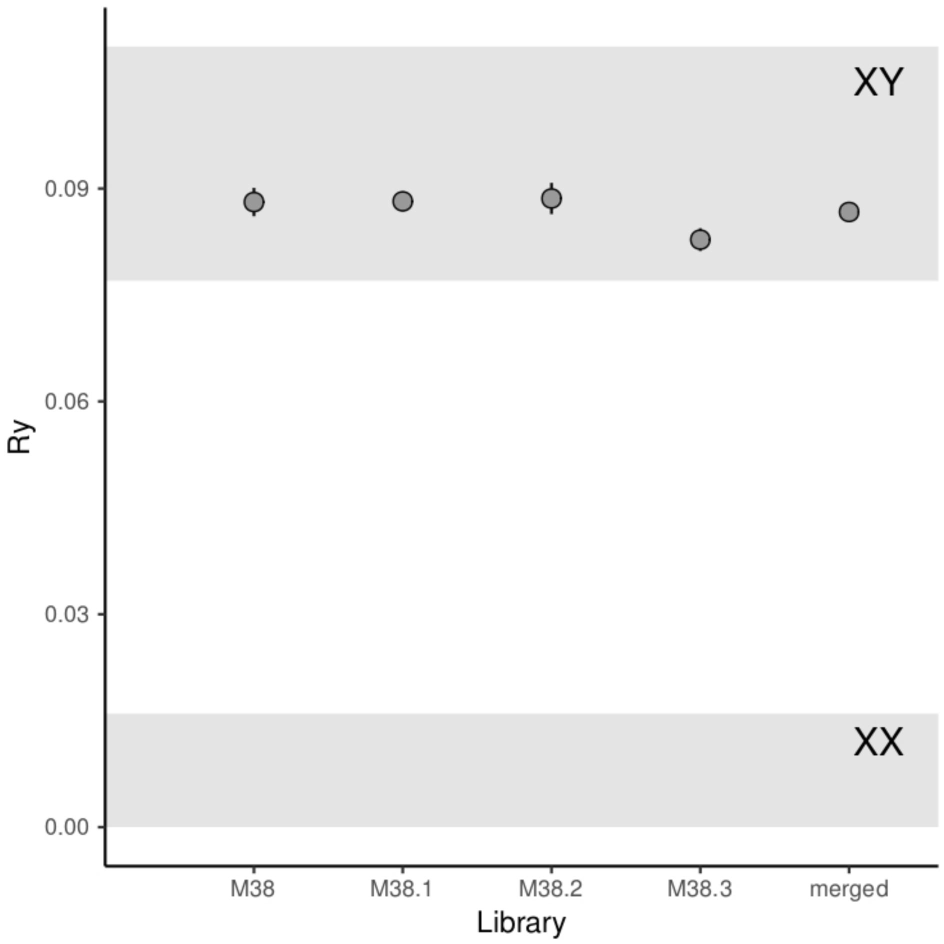

Supplementary Figure S3. Sex assignment plot (error bars represent 95% confidence intervals). On the x-axis the name of each library for UE2298/MS060, on the y-axis the *Ry* score used to determine the genetic sex.

Supplementary Figure S4. Most-parsimonious phylogeny of mtDNA haplogroup U6 (Excel file).

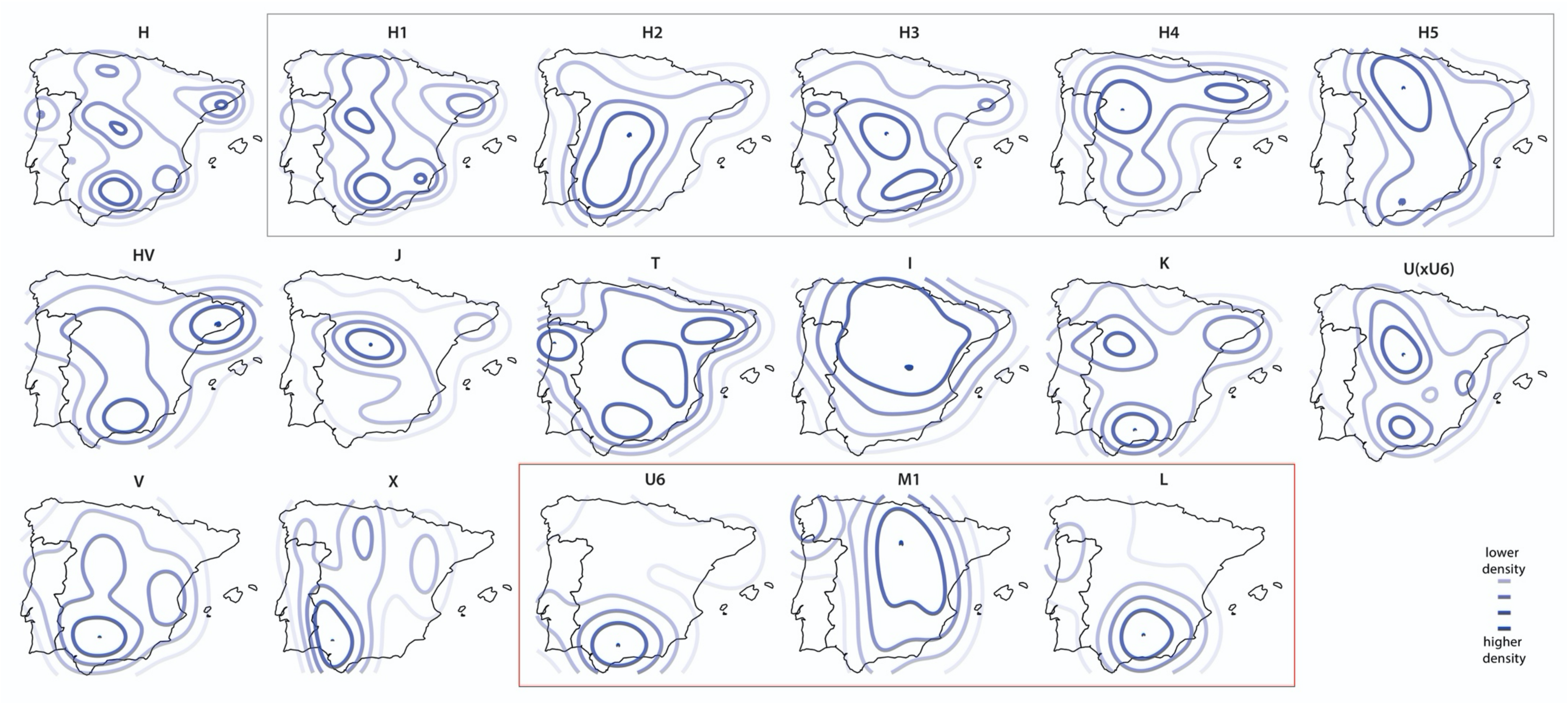

Supplementary Figure S5. Density maps of different mtDNA haplogroups in present-day Iberia (haplogroup H main subclades inside grey box, African-related haplogroups inside red box).

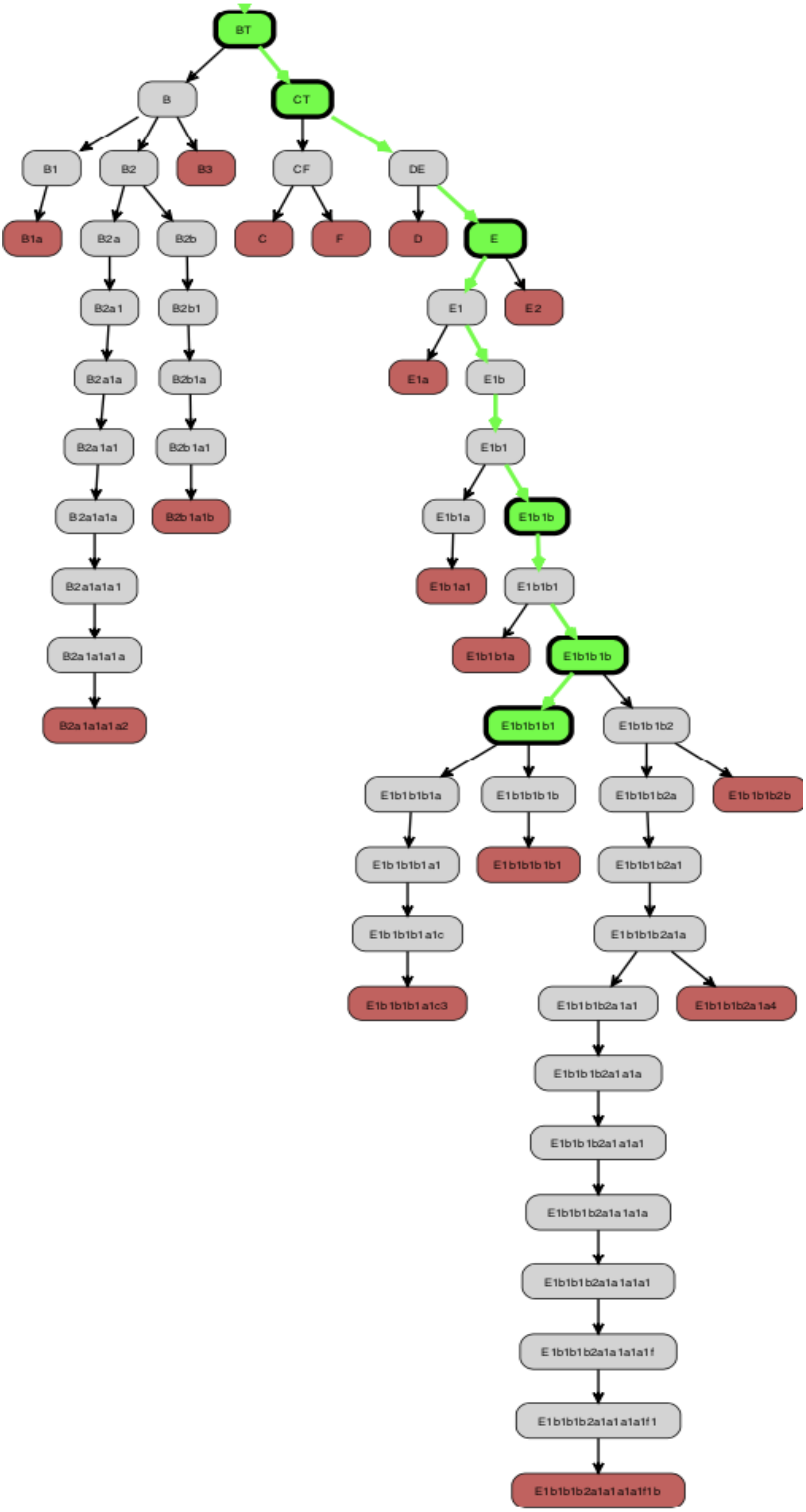
BT
B
CT
B1
B2
B3
CF
DE
B1a
B2a
B2b
C
F
D
E
B2a1
B2b1
E1
E2
B2a1a
B2b1a
E1a
E1b
B2a1a1
B2b1a1
E1b1
B2a1a1a
B2b1a1b
E1b1a
E1b1b
B2a1a1a1
E1b1a1
E1b1b1
B2a1a1a1a
E1b1b1a
E1b1b1b
B2a1a1a1a2
E1b1b1b1
E1b1b1b2
E1b1b1b1a
E1b1b1b1b
E1b1b1b2a
E1b1b1b2b
E1b1b1b1a1
E1b1b1b1b1
E1b1b1b2a1
E1b1b1b1a1c
E1b1b1b2a1a
E1b1b1b1a1c3
E1b1b1b2a1a1
E1b1b1b2a1a4
E1b1b1b2a1a1a
E1b1b1b2a1a1a1
E1b1b1b2a1a1a1a
E1b1b1b2a1a1a1a1
E1b1b1b2a1a1a1a1f
E1b1b1b2a1a1a1a1f1
E1b1b1b2a1a1a1a1f1b

Supplementary Figure S6. PathPhynder decision tree using only the SNPs in the ISOGG 2018 database. Coloured labels indicate the allele status at markers defining each haplogroup (green for derived allele, red for ancestral and grey for missing positions). UE2298/MS060 can be assigned to the E1b1b1b1 haplogroup but lacks sufficient data for a more downstream assignment.

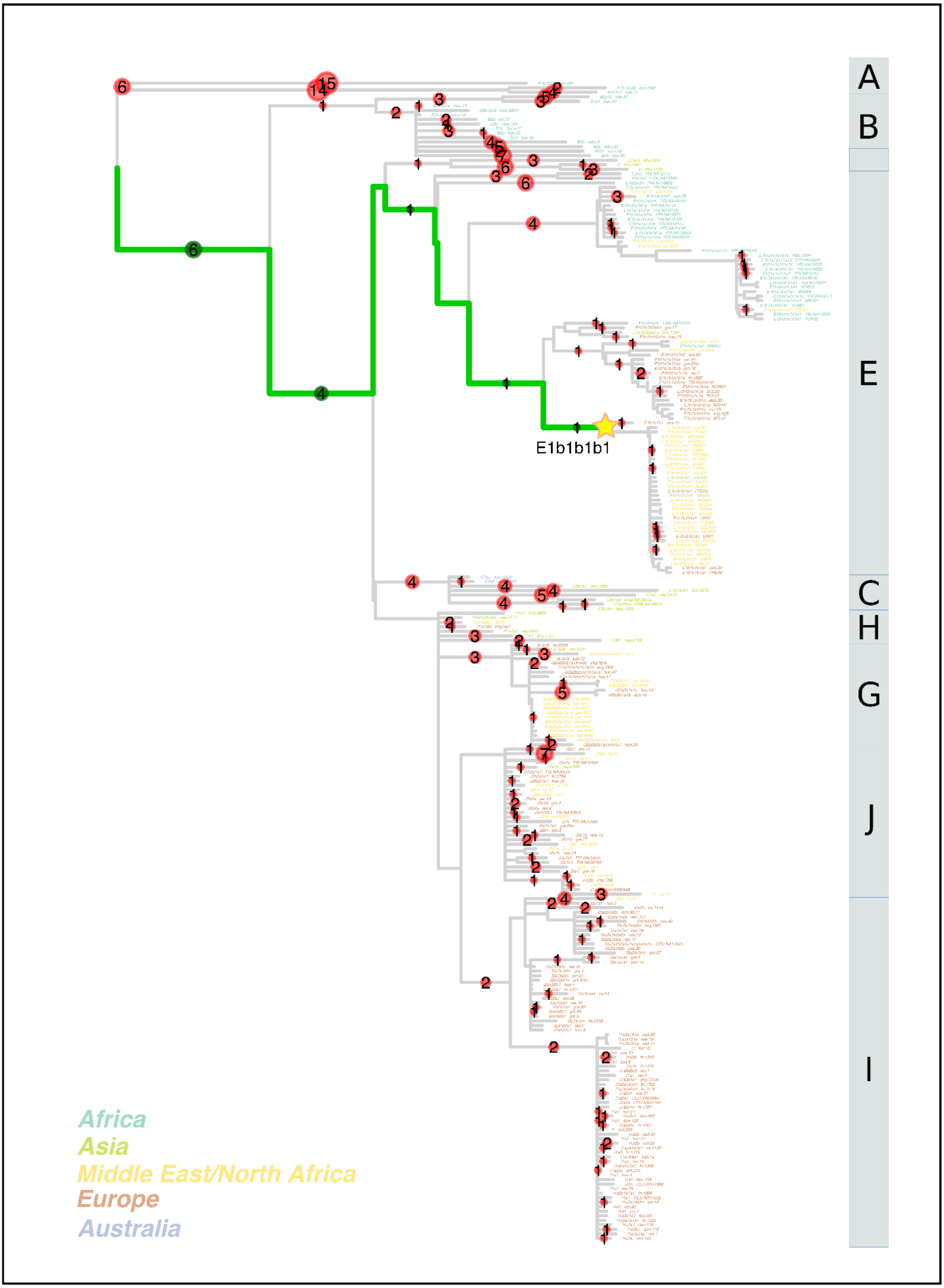

A
B
E
C
H
G
J
I
E1b1b1b1
Africa
Asia
Middle East/North Africa
Europe
Australia

Supplementary Figure S7. Neighbour-joining phylogenetic tree estimated with 256 Y-chromosome sequences from worldwide populations[26,27]. Coloured circles indicate the number of derived (green) or ancestral (red) branch defining markers identified in the ancient individual. The branches coloured in green indicate the path with greatest support for the inclusion of UE2298/MS060 within a clade (star), which contains Spanish, Middle Eastern and North African individuals belonging to the E–M310 (E1b1b1b1) Y-chromosome lineage.

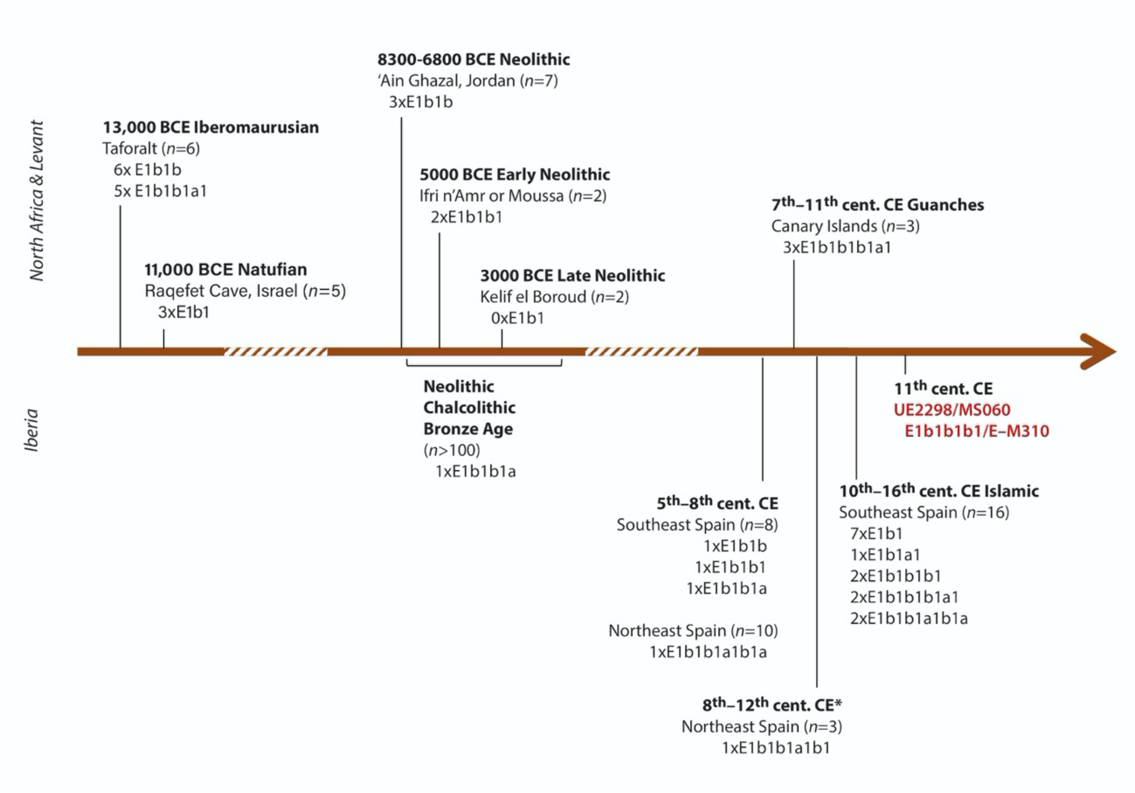

Supplementary Figure S8. Timeline showing occurrence of Y-chromosome E1b1 lineages in the archaeological record of Spain, North Africa and Levant through time[31,85,87,89,117–119]. Population from Northeast Spain dating to 8th–12th centuries CE (marked with *) corresponds to the archaeological site of Sant Julià de Ramis, where North African–related ancestry has been identified before, following a short period of Islamic influence[33].

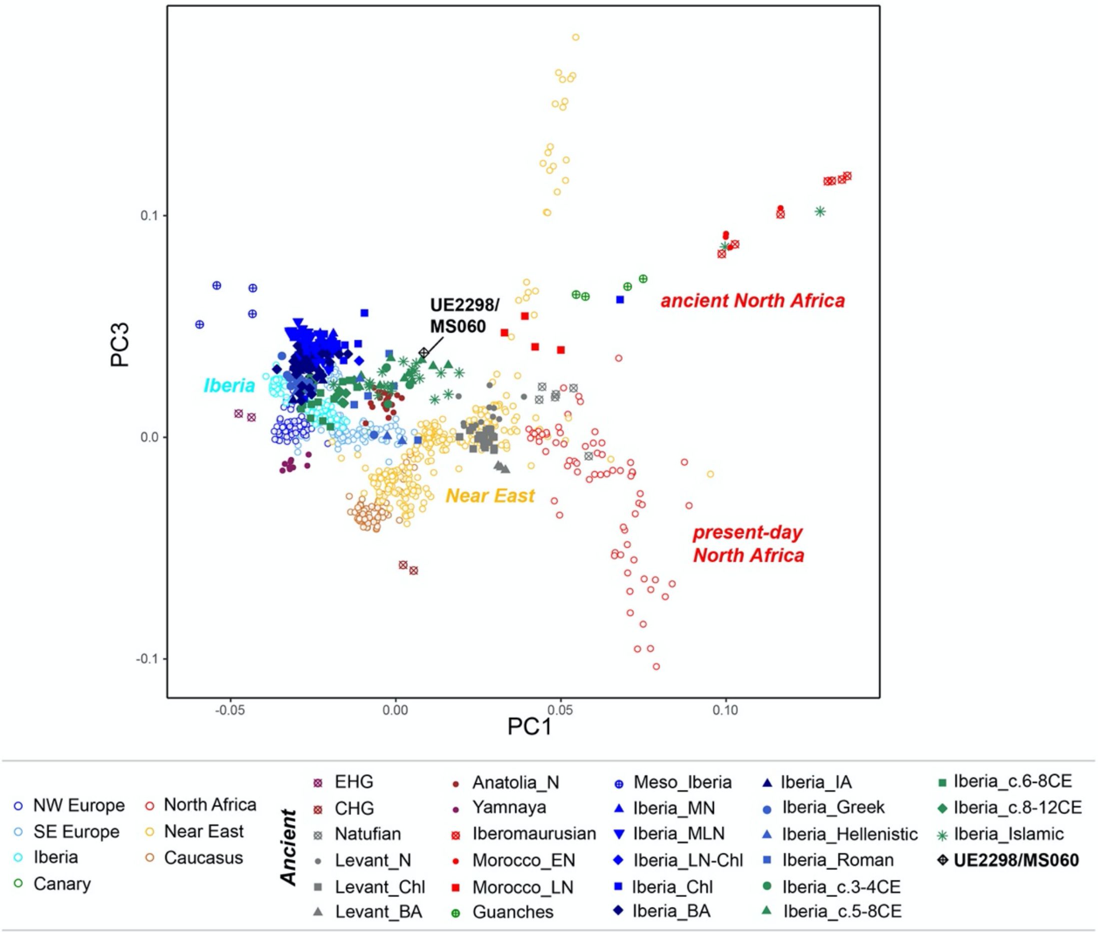

Supplementary Figure S9. PCA (PC1 and PC3) projecting 336 ancient samples on 702 modern individuals from North African, European, Near Eastern and Caucasian populations. Abbreviations as follows: E/CHG – Eastern/Caucasus Hunter-Gatherers, Meso – Mesolithic, (E/M/L) N – (Early/Middle/Late) Neolithic, Chl – Chalcolithic, BA – Bronze Age, IA – Iron Age, c. – centuries.

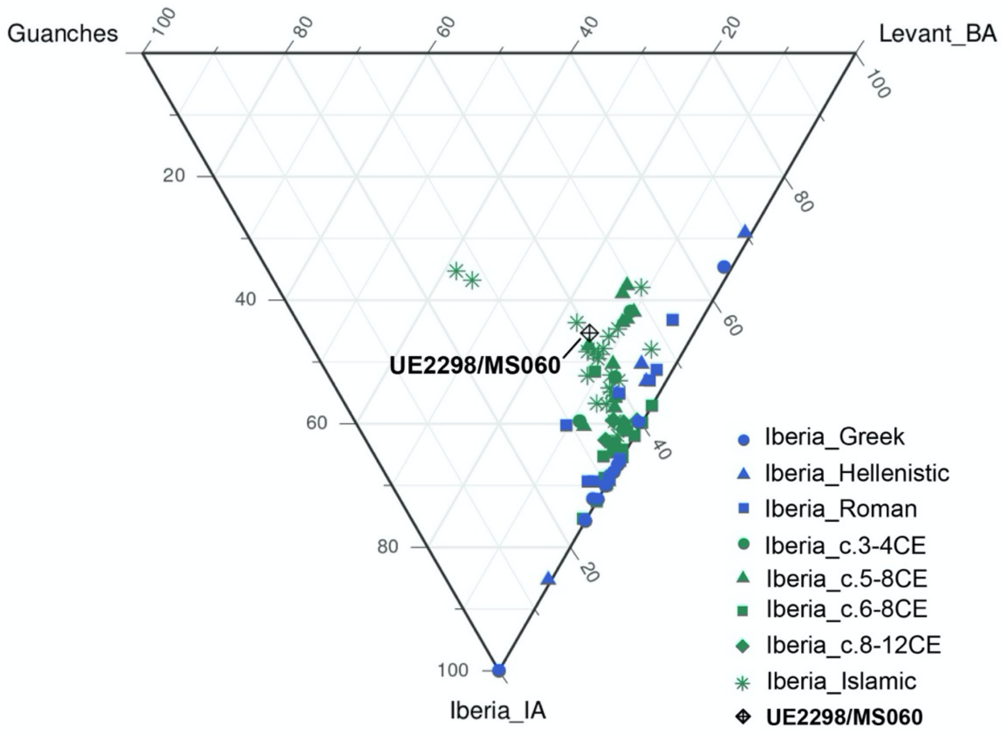

Supplementary Figure S10. Ternary Plot showing supervised ADMIXTURE proportions (*K*=3) of post-Iron Age individuals, using *Iberia_IA*, *Guanches* and *Levant_BA* as reference populations. Abbreviations as in Figure 3 and Supplementary Figure S9.

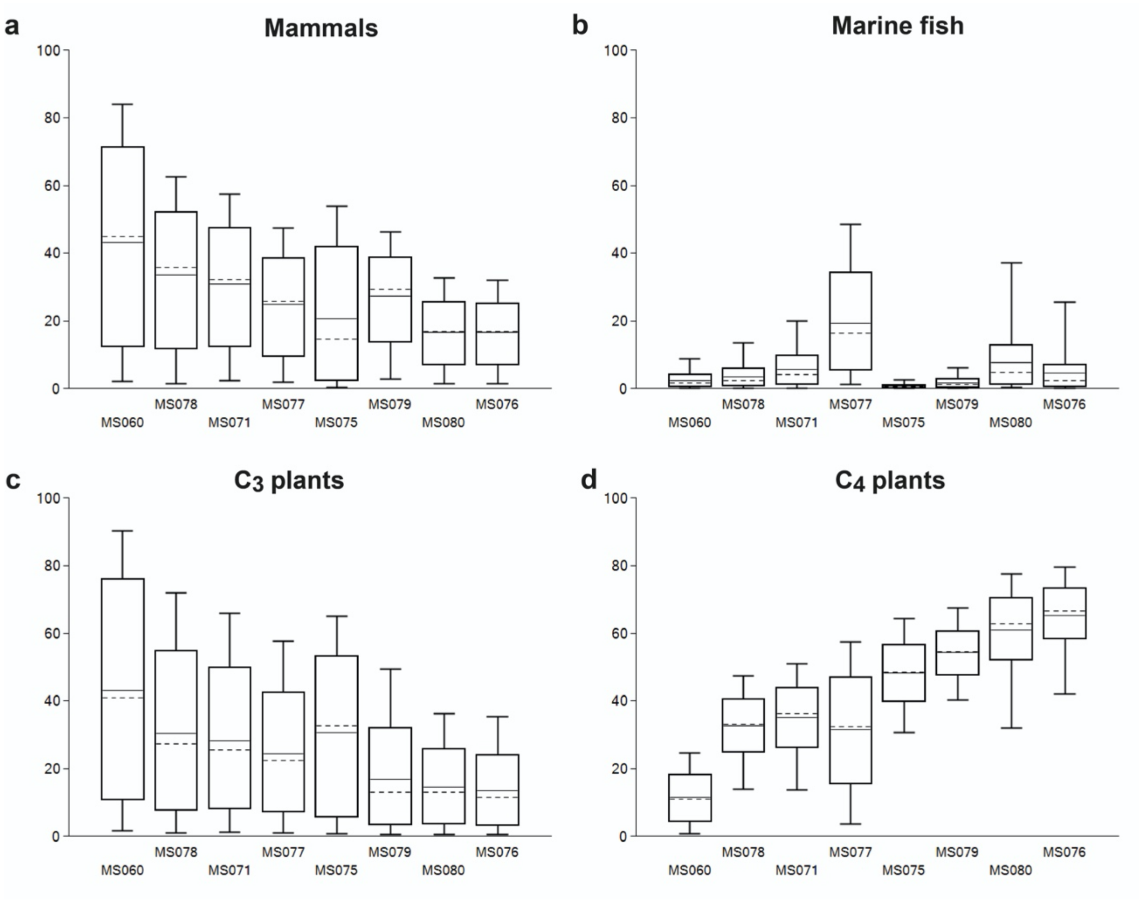

Supplementary Figure S11. Proportions of **(a)** terrestrial mammals, **(b)** marine fish, **(c)** C_3 plants and **(d)** C_4 plants intake for each of the individuals analysed, calculated using FRUITS[59].

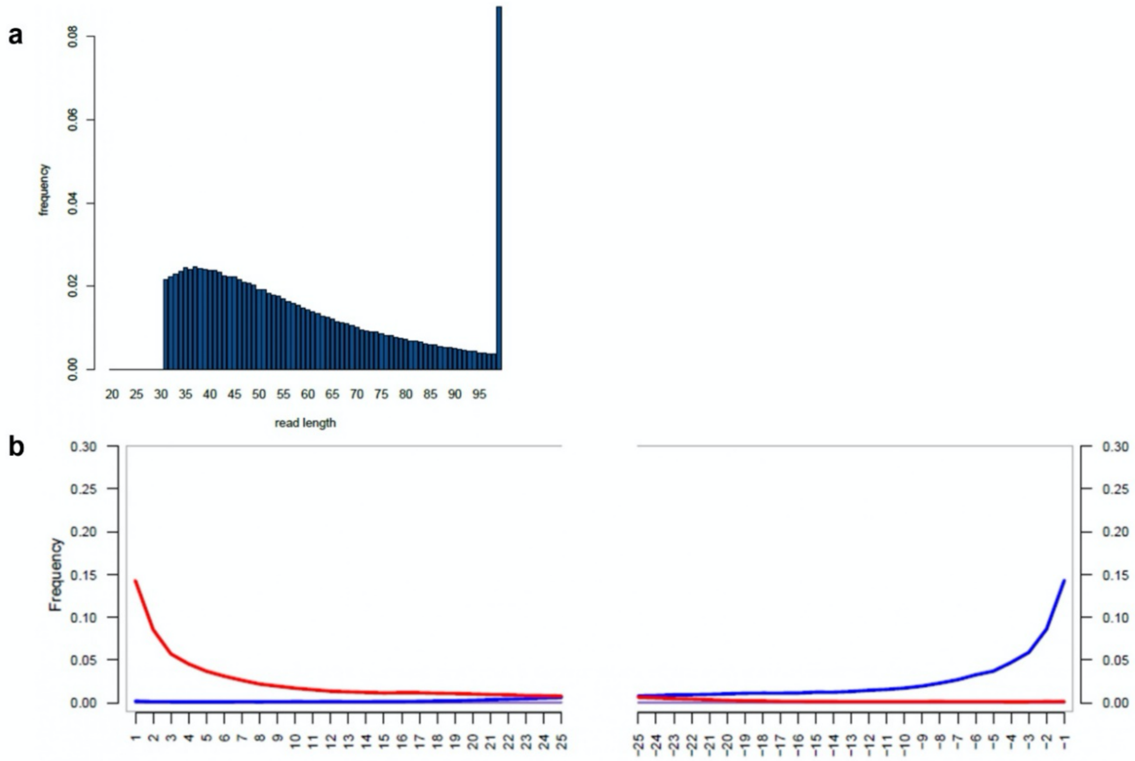

Supplementary Figure S12. a) Read length distribution (plot from bamdamage). **b)** Damage pattern showing the typical C>T (5'end) and G>A (3'end) misincorporations in the non-USER-treated library, confirming authenticity of aDNA (plot from mapDamage).

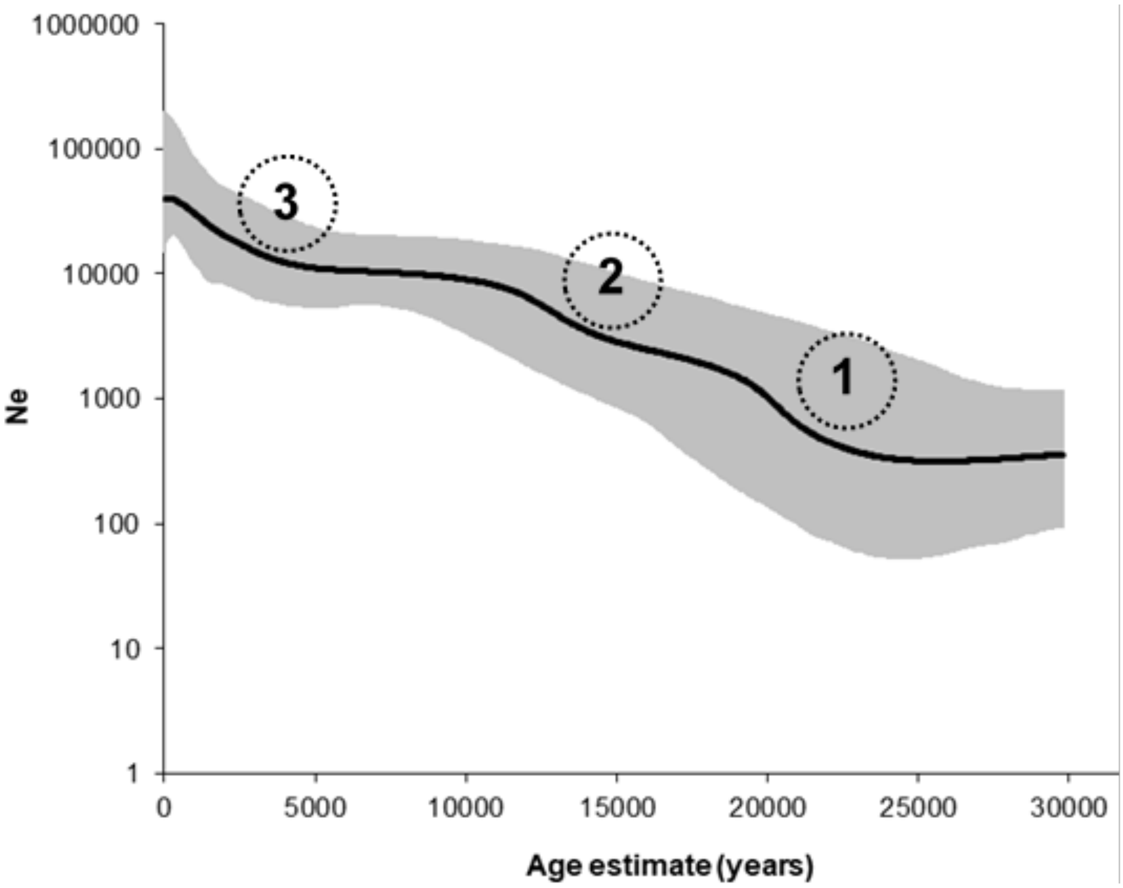

Supplementary Figure S13. BSP indicating the median of the *Ne* associated to mtDNA haplogroup U6 through time. Three main moments of population increment: (1) around the LGM, just before 20 ka; (2) in the Late Glacial (12–15 ka) period; and (3) in the last 5 ka, with an acceleration in the last ~2 ka.

References

1. Barrachina, A. La necròpolis islàmica de la plaça de l'Almudín, Sogorb (Alt Palància). Estudi antropològic i cronològic. *Quad. prehistòria i Arqueol. Castelló* **24**, 281–294 (2004).
2. Forner, A. Estudio antropológico y paleopatológico de un individuo de la necrópolis del Almudín. (Universitat de València, 2002).
3. Yang, D. Y., Eng, B., Waye, J. S., Dudar, J. C. & Saunders, S. R. Technical note: Improved DNA extraction from ancient bones using silica- based spin columns. *Am. J. Phys. Anthropol.* **105**, 539–543 (1998).
4. MacHugh, D., Edwards, C., Bailey, J., Bancroft, D. & Bradley, D. The extraction and analysis of ancient DNA from bone and teeth: a survey of current methodologies. *Anc. Biomol.* **3**, 81–102 (2000).
5. Meyer, M. & Kircher, M. Illumina sequencing library preparation for highly multiplexed target capture and sequencing. *Cold Spring Harb. Protoc.* **2010**, pdb.prot5448; 10.1101/pdb.prot5448 (2010).
6. Gamba, C. *et al.* Genome flux and stasis in a five millennium transect of European prehistory. *Nat. Commun.* **5**, 5257; 10.1038/ncomms6257 (2014).
7. Cassidy, L. M. *et al.* Neolithic and Bronze Age migration to Ireland and establishment of the insular Atlantic genome. *Proc. Natl. Acad. Sci. U. S. A.* **113**, 368–373 (2016).
8. Andrews, S. FastQC: a quality control tool for high throughput sequence data. (2010). Available at: http://www.bioinformatics.babraham.ac.uk/projects/fastqc
9. Renaud, G., Stenzel, U. & Kelso, J. leeHom: adaptor trimming and merging for Illumina sequencing reads. *Nucleic Acids Res.* **42**, e141; 10.1093/nar/gku699 (2014).
10. Li, H. Aligning sequence reads, clone sequences and assembly contigs with BWA-MEM. *arXiv*; arxiv.org/abs/1303.3997 (2013).
11. Schubert, M. *et al.* Improving ancient DNA read mapping against modern reference genomes. *BMC Genomics* **13**, 178; 10.1186/1471-2164-13-178 (2012).
12. Li, H. *et al.* The Sequence Alignment/Map format and SAMtools. *Bioinformatics* **25**, 2078–2079 (2009).
13. Okonechnikov, K., Conesa, A. & García-Alcalde, F. Qualimap 2: advanced multi-sample quality control for high-throughput sequencing data. *Bioinformatics* **32**, 292–294 (2015).
14. Renaud, G., Slon, V., Duggan, A. T. & Kelso, J. Schmutzi: estimation of contamination and endogenous mitochondrial consensus calling for ancient DNA. *Genome Biol.* **16**, 224; 10.1186/s13059-015-0776-0 (2015).
15. Skoglund, P., Storå, J., Götherström, A. & Jakobsson, M. Accurate sex identification of ancient human remains using DNA shotgun sequencing. *J. Archaeol. Sci.* **40**, 4477–4482 (2013).

16. Korneliussen, T. S., Albrechtsen, A. & Nielsen, R. ANGSD: Analysis of Next Generation Sequencing Data. *BMC Bioinformatics* **15**, 356; 10.1186/s12859-014-0356-4 (2014).
17. Jónsson, H., Ginolhac, A., Schubert, M., Johnson, P. L. F. & Orlando, L. mapDamage2.0: fast approximate Bayesian estimates of ancient DNA damage parameters. *Bioinformatics* **29**, 1682–1684 (2013).
18. Malaspinas, A.-S. *et al.* bammds: a tool for assessing the ancestry of low-depth whole-genome data using multidimensional scaling (MDS). *Bioinformatics* **30**, 2962–2964 (2014).
19. Jun, G., Wing, M. K., Abecasis, G. R. & Kang, H. M. An efficient and scalable analysis framework for variant extraction and refinement from population-scale DNA sequence data. *Genome Res*. **25**, 918–925 (2015).
20. McKenna, A. *et al.* The Genome Analysis Toolkit: a MapReduce framework for analyzing next-generation DNA sequencing data. *Genome Res.* **20**, 1297–1303 (2010).
21. Kloss-Brandstätter, A. *et al.* HaploGrep: a fast and reliable algorithm for automatic classification of mitochondrial DNA haplogroups. *Hum. Mutat.* **32**, 25–32 (2011).
22. van Oven, M. PhyloTree Build 17: Growing the human mitochondrial DNA tree. *Forensic Sci. Int. Genet. Suppl. Ser.* **5**, e392–e394 (2015).
23. Thorvaldsdottir, H., Robinson, J. T. & Mesirov, J. P. Integrative Genomics Viewer (IGV): high-performance genomics data visualization and exploration. *Brief. Bioinform.* **14**, 178–192 (2013).
24. Ralf, A., Montiel González, D., Zhong, K. & Kayser, M. Yleaf: Software for human Y-chromosomal haplogroup inference from next-generation sequencing data. *Mol. Biol. Evol.* **35**, 1291–1294 (2018).
25. Martiniano, R., De Sanctis, B., Hallast, P. & Durbin, R. Placing ancient DNA sequences into reference phylogenies. *bioRxiv*; 10.1101/2020.12.19.423614 (2020).
26. Hallast, P. *et al.* The Y-chromosome tree bursts into leaf: 13,000 high-confidence SNPs covering the majority of known clades. *Mol. Biol. Evol.* **32**, 661–673 (2015).
27. Solé-Morata, N. *et al.* Whole Y-chromosome sequences reveal an extremely recent origin of the most common North African paternal lineage E-M183 (M81). *Sci. Rep.* **7**, 15941; 10.1038/s41598-017-16271-y (2017).
28. Paradis, E. & Schliep, K. ape 5.0: an environment for modern phylogenetics and evolutionary analyses in R. *Bioinformatics* **35**, 526–528 (2018).
29. The R Development Core Team. R: A Language and Environment for Statistical Computing (2008). Accessible at: https://www.R-project.org.
30. Patterson, N., Price, A. L. & Reich, D. Population structure and eigenanalysis. *PLoS Genet.* **2**, e190; 10.1371/journal.pgen.0020190 (2006).
31. Lazaridis, I. *et al.* Genomic insights into the origin of farming in the ancient Near East. *Nature* **536**, 419–424 (2016).

32. Purcell, S. *et al.* PLINK: a tool set for whole-genome association and population-based linkage analyses. *Am. J. Hum. Genet.* **81**, 559–575 (2007).
33. Olalde, I. *et al.* The genomic history of the Iberian Peninsula over the past 8000 years. *Science.* **363**, 1230–1234 (2019).
34. Alexander, D. H., Novembre, J. & Lange, K. Fast model-based estimation of ancestry in unrelated individuals. *Genome Res.* **19**, 1655–1664 (2009).
35. Fu, Q. *et al.* Genome sequence of a 45,000-year-old modern human from western Siberia. *Nature* **514**, 445–449 (2014).
36. Patterson, N. *et al.* Ancient admixture in human history. *Genetics* **192**, 1065–1093 (2012).
37. Haak, W. *et al.* Massive migration from the steppe was a source for Indo-European languages in Europe. *Nature* 207–211 (2015).
38. Wickham, H. *ggplot2: Elegant Graphics for Data Analysis*. (Springer, 2016).
39. RStudio Team. RStudio: Integrated Development for R (2020). Accessible at: http://www.rstudio.com.
40. Barral-Arca, R. *et al*. Meta-analysis of mitochondrial DNA variation in the Iberian Peninsula. *PLoS One* **11**, e0159735; 10.1371/journal.pone.0159735 (2016).
41. Miller, S. A., Dykes, D. D. & Polesky, H. F. A simple salting out procedure for extracting DNA from human nucleated cells. *Nucleic Acids Res.* **16**, 1215; 10.1093/nar/16.3.1215 (1988).
42. Brandini, S. *et al.* The Paleo-Indian entry into South America according to mitogenomes. *Mol. Biol. Evol.* **35**, 299–311 (2018).
43. Peltzer, A. *et al.* EAGER: efficient ancient genome reconstruction. *Genome Biol.* **17**, 60 (2016).
44. Schubert, M., Lindgreen, S. & Orlando, L. AdapterRemoval v2: rapid adapter trimming, identification, and read merging. *BMC Res. Notes* **9**, 88 (2016).
45. Danecek, P. *et al.* The variant call format and VCFtools. *Bioinformatics* **27**, 2156–2158 (2011).
46. Holle, R., Happich, M., Löwel, H. & Wichmann, H. E. KORA - A research platform for population based health research. *Gesundheitswesen* **67**, S19–25 (2005).
47. Soares, P. *et al.* Correcting for purifying selection: an improved human mitochondrial molecular clock. *Am. J. Hum. Genet.* **84**, 740–759 (2009).
48. Saillard, J., Forster, P., Lynnerup, N. & Nørby, S. mtDNA Variation among Greenland Eskimos: The Edge of the Beringian Expansion. *Am. J. Hum. Genet.* **1**, 718–726 (2000).
49. Yang, Z. PAML: a program package for phylogenetic analysis by maximum likelihood. *Comput. Appl. Biosci. CABIOS* **13**, 555–556 (1997).
50. Drummond, A. J., Rambaut, A., Shapiro, B. & Pybus, O. G. Bayesian Coalescent Inference of Past Population Dynamics from Molecular Sequences. *Mol Biol Evol* **22**, 1185–1192

(2005).
51. Drummond, A. J., Suchard, M. A., Xie, D. & Rambaut, A. Bayesian phylogenetics with BEAUti and the BEAST 1.7. *Mol. Biol. Evol.* **29**, 1969–1973 (2012).
52. Pereira, L. *et al.* Population expansion in the North African late Pleistocene signalled by mitochondrial DNA haplogroup U6. *BMC Evol. Biol.* **10**, 390; 10.1186/1471-2148-10-390 (2010).
53. Moorjani, P. *et al.* A genetic method for dating ancient genomes provides a direct estimate of human generation interval in the last 45,000 years. *Proc. Natl. Acad. Sci. U. S. A.* **113**, 5652–5657 (2016).
54. Britton, K., Fuller, B. T., Tütken, T., Mays, S. & Richards, M. P. Oxygen isotope analysis of human bone phosphate evidences weaning age in archaeological populations. *Am. J. Phys. Anthropol.* **157**, 226–241 (2015).
55. Quandt, S. A. Breasts, Bottles, and Babies: A History of Infant Feeding. Valerie Fildes. *Med. Anthropol. Q.* **2**, 306–308 (1988).
56. Ventresca Miller, A. *et al.* Sampling and pretreatment of tooth enamel carbonate for stable carbon and oxygen isotope analysis. *J. Vis. Exp.* **2018**, e58002; 10.3791/58002 (2018).
57. Alexander, M. M., Gerrard, C. M., Gutiérrez, A. & Millard, A. R. Diet, society, and economy in late medieval Spain: Stable isotope evidence from Muslims and Christians from Gandía, Valencia. *Am. J. Phys. Anthropol.* **156**, 263–273 (2015).
58. Alexander, M. M., Gutiérrez, A., Millard, A. R., Richards, M. P. & Gerrard, C. M. Economic and socio-cultural consequences of changing political rule on human and faunal diets in medieval Valencia (c. fifth–fifteenth century AD) as evidenced by stable isotopes. *Archaeol. Anthropol. Sci.* **11**, 3875–3893 (2019).
59. Fernandes, R., Millard, A. R., Brabec, M., Nadeau, M.-J. & Grootes, P. Food Reconstruction Using Isotopic Transferred Signals (FRUITS): A Bayesian model for diet reconstruction. *PLoS One* **9**, e87436; 0.1371/journal.pone.0087436 (2014).
60. Cheung, C. & Szpak, P. interpreting past human diets using stable isotope mixing models. *J. Archaeol. Method Theory*; 10.1007/s10816-020-09492-5 (2020).
61. Fernández-Crespo, T., Ordoño, J., Bogaard, A., Llanos, A. & Schulting, R. A snapshot of subsistence in Iron Age Iberia: The case of La Hoya village. *J. Archaeol. Sci. Reports* **28**, 102037; 10.1016/j.jasrep.2019.102037 (2019).
62. Fernandes, R., Grootes, P., Nadeau, M.-J. & Nehlich, O. Quantitative diet reconstruction of a Neolithic population using a Bayesian mixing model (FRUITS): The case study of Ostorf (Germany). *Am. J. Phys. Anthropol.* **158**, 325–340 (2015).
63. Fernandes, R., Nadeau, M. J. & Grootes, P. M. Macronutrient-based model for dietary carbon routing in bone collagen and bioapatite. *Archaeol. Anthropol. Sci.* **4**, 291–301 (2012).
64. Buckley, M., Collins, M., Thomas-Oaies, J. & Wilson, J. C. Species identification by

analysis of bone collagen using matrix-assisted laser desorption/ionisation time-of-flight mass spectrometry. *Rapid Commun. Mass Spectrom.* **23**, 3843–3854 (2009).

65. Buckley, M. *et al.* Distinguishing between archaeological sheep and goat bones using a single collagen peptide. *J. Archaeol. Sci.* **37**, 13–20 (2010).
66. Strohalm, M., Hassman, M., Košata, B. & Kodíček, M. mMass data miner: An open source alternative for mass spectrometric data analysis. *Rapid Communications in Mass Spectrometry* **22**, 905–908 (2008).
67. Kirby, D. P., Buckley, M., Promise, E., Trauger, S. A. & Holdcraft, T. R. Identification of collagen-based materials in cultural heritage. *Analyst* **138**, 4849–4858 (2013).
68. Welker, F. *et al.* Palaeoproteomic evidence identifies archaic hominins associated with the Châtelperronian at the Grotte du Renne. *Proc. Natl. Acad. Sci. U. S. A.* **113**, 11162–11167 (2016).
69. Malyarchuk, B. *et al.* The peopling of Europe from the mitochondrial haplogroup U5 perspective. *PLoS One* **5**, e10285; 10.1371/journal.pone.0010285 (2010).
70. Brandt, G. *et al.* Ancient DNA Reveals Key Stages in the Formation of Central European Mitochondrial Genetic Diversity. *Science* **342**, 257–261 (2013).
71. Malyarchuk, B. *et al.* Mitochondrial DNA phylogeny in Eastern and Western Slavs. *Mol. Biol. Evol.* **25**, 1651–1658 (2008).
72. Costa, M. D. *et al.* A substantial prehistoric European ancestry amongst Ashkenazi maternal lineages. *Nat. Commun.* **4**, 2543; 10.1038/ncomms3543 (2013).
73. Al-Zahery, N. *et al.* In search of the genetic footprints of Sumerians: a survey of Y-chromosome and mtDNA variation in the Marsh Arabs of Iraq. *BMC Evol. Biol.* **11**, 288; 10.1186/1471-2148-11-288 (2011).
74. Derenko, M. *et al.* Complete mitochondrial DNA diversity in Iranians. *PLoS One* **8**, e80673; 10.1371/journal.pone.0080673 (2013).
75. Sahakyan, H. *et al.* Origin and spread of human mitochondrial DNA haplogroup U7. *Sci. Rep.* **7**, 46044; 10.1038/srep46044 (2017).
76. Metspalu, M. *et al.* Most of the extant mtDNA boundaries in south and southwest Asia were likely shaped during the initial settlement of Eurasia by anatomically modern humans. *BMC Genet.* **5**, 26; 10.1186/1471-2156-5-26 (2004).
77. Hervella, M. *et al.* The mitogenome of a 35,000-year-old Homo sapiens from Europe supports a Palaeolithic back-migration to Africa. *Sci. Rep.* **6**, 25501; 10.1038/srep25501 (2016).
78. Fu, Q. *et al.* The genetic history of Ice Age Europe. *Nature* **534**, 200–205 (2016).
79. Svensson, E. *et al.* Genome of Peştera Muierii skull shows high diversity and low mutational load in pre-glacial Europe. *Curr. Biol.* In press;10.1016/j.cub.2021.04.045 (2021).
80. Lazaridis, I. *et al.* Paleolithic DNA from the Caucasus reveals core of West Eurasian

ancestry. *BioRxiv;* 10.1101/423079 (2018).

81. Macaulay, V. *et al.* The emerging tree of West Eurasian mtDNAs: a synthesis of control-region sequences and RFLPs. *Am J Hum Genet* **64**, 232–249 (1999).
82. Olivieri, A. *et al.* The mtDNA legacy of the Levantine Early Upper Palaeolithic in Africa. *Science.* **314**, 1767–1770 (2006).
83. Henn, B. M. *et al.* Genomic ancestry of North Africans supports back-to-Africa migrations. *PLoS Genet.* **8**, e1002397; 10.1371/journal.pgen.1002397 (2012).
84. Sánchez-Quinto, F. *et al.* North African populations carry the signature of admixture with Neandertals. *PLoS One* **7**, e47765; 10.1371/journal.pone.0047765 (2012).
85. van de Loosdrecht, M. *et al.* Pleistocene North African genomes link Near Eastern and sub-Saharan African human populations. *Science* **360**, 548–552 (2018).
86. Hogue, J. T. & Barton, R. N. E. New radiocarbon dates for the earliest Later Stone Age microlithic technology in Northwest Africa. *Quat. Int.* **413**, 62–75 (2016).
87. Fregel, R. *et al.* Ancient genomes from North Africa evidence prehistoric migrations to the Maghreb from both the Levant and Europe. *Proc. Natl. Acad. Sci. U. S. A.* **115**, 6774–6779 (2018).
88. Arauna, L. R. *et al.* Recent historical migrations have shaped the gene pool of Arabs and Berbers in North Africa. *Mol. Biol. Evol.* **34**, 318–329 (2016).
89. Rodríguez-Varela, R. *et al.* Genomic analyses of pre-European conquest human Remains from the Canary Islands reveal close affinity to modern North Africans. *Curr. Biol.* **27**, 3396-3402.e5; 10.1016/j.cub.2017.09.059 (2017).
90. Maca-Meyer, N. *et al.* Ancient mtDNA analysis and the origin of the Guanches. *Eur. J. Hum. Genet.* **12**, 155–162 (2004).
91. Secher, B. *et al.* The history of the North African mitochondrial DNA haplogroup U6 gene flow into the African, Eurasian and American continents. *BMC Evol. Biol.* **14**, 109; 10.1186/1471-2148-14-109 (2014).
92. QEN members. *Review and atlas of palaeovegetation: Preliminary land ecosystem maps of the world since the Last Glacial Maximum* (eds. Adams J.M. & Faure H.) (Oak Ridge National Laboratory, 2017). Available at http://www.esd.ornl.gov/projects/qen/adams1.html
93. Shanahan, T. M. *et al.* The time-transgressive termination of the African humid period. *Nat. Geosci.* **8**, 140–144 (2015).
94. Otto-Bliesner, B. L. *et al.* Coherent changes of southeastern equatorial and northern African rainfall during the last deglaciation. *Science.* **346**, 1223–1227 (2014).
95. Nicoll, K. Recent environmental change and prehistoric human activity in Egypt and Northern Sudan. *Quat. Sci. Rev.* **23**, 561–580 (2004).
96. Jousse, H. What is the impact of Holocene climatic changes on human societies? Analysis of West African Neolithic populations dietary customs. *Quat. Int.* **151**, 63–73 (2006).

97. Kuper, R. & Kröpelin, S. Climate-controlled Holocene occupation in the Sahara: Motor of Africa's evolution. *Science.* **313**, 803–807 (2006).

98. Lightfoot, E. & O'Connell, T. C. On the use of biomineral oxygen isotope data to identify human migrants in the archaeological record: Intra-sample variation, statistical methods and geographical considerations. *PLoS One* **11**, e0153850; 10.1371/journal.pone.0153850 (2016).

99. Coplen, T. B., Kendall, C. & Hopple, J. Comparison of stable isotope reference samples. *Nature* **302**, 236–238 (1983).

100. Chenery, C. A., Pashley, V., Lamb, A. L., Sloane, H. J. & Evans, J. A. The oxygen isotope relationship between the phosphate and structural carbonate fractions of human bioapatite. *Rapid Commun. Mass Spectrom.* **26**, 309–319 (2012).

101. Daux, V. et al. Oxygen isotope fractionation between human phosphate and water revisited. *J. Hum. Evol.* **55**, 1138–1147 (2008).

102. Pederzani, S. & Britton, K. Oxygen isotopes in bioarchaeology: Principles and applications, challenges and opportunities. *Earth-Science Rev.* **188**, 77–107 (2019).

103. IAEA/WMO. Global network of isotopes in precipitation. The GNIP database. Accessible at: https://nucleus.iaea.org/wiser. (2019).

104. Brettell, R., Montgomery, J. & Evans, J. Brewing and stewing: The effect of culturally mediated behaviour on the oxygen isotope composition of ingested fluids and the implications for human provenance studies. *J. Anal. At. Spectrom.* **27**, 778–785 (2012)

105. White, C., Longstaffe, F. J. & Law, K. R. Exploring the effects of environment, physiology and diet on oxygen isotope ratios in ancient Nubian bones and teeth. *J. Archaeol. Sci.* **31**, 233–250 (2004).

106. Stantis, C. *et al.* Multi-isotopic study of diet and mobility in the northeastern Nile Delta. *Archaeol. Anthropol. Sci.* **13**, 105; 10.1007/s12520-021-01344-x (2021).

107. Bowen, G. J. & Revenaugh, J. Interpolating the isotopic composition of modern meteoric precipitation. *Water Resour. Res.* **39**, 1299; 10.1029/2003WR002086 (2003).

108. Turner, B. L., Edwards, J. L., Quinn, E. A., Kingston, J. D. & Van Gerven, D. P. Age-related variation in isotopic indicators of diet at medieval Kulubnarti, Sudanese Nubia. *Int. J. Osteoarchaeol.* **17**, 1–25 (2007).

109. Planillo Portolés, J. Á. El camino de pescadores. Una ruta comercial entre Valldecrist y el Mediterráneo. *Orleyl Rev. l'Associació Arqueol. la Vall d'Uixó* (2018).

110. Salazar-García, D. C., Richards, M. P., Nehlich, O. & Henry, A. G. Dental calculus is not equivalent to bone collagen for isotope analysis: a comparison between carbon and nitrogen stable isotope analysis of bulk dental calculus, bone and dentine collagen from same individuals from the Medieval site of El Raval (Alicante, Spain). *J. Archaeol. Sci.* **47**, 70–77 (2014).

111. Guede, I. *et al.* Isotope analyses to explore diet and mobility in a medieval Muslim population at Tauste (NE Spain). *PLoS One* **12**, e0176572; 10.1371/journal.pone.0176572 (2017).
112. Richards, M. P. & Hedges, R. E. M. Stable isotope evidence for similarities in the types of marine foods used by late Mesolithic humans at sites along the Atlantic coast of Europe. *J. Archaeol. Sci.* **26**, 717–722 (1999).
113. Craig, O. E. *et al.* Stable isotopic evidence for diet at the Imperial Roman coastal site of Velia (1st and 2nd Centuries AD) in Southern Italy. *Am. J. Phys. Anthropol.* **139**, 572–583 (2009).
114. Dury, G. *et al.* The Islamic cemetery at 33 Bartomeu Vicent Ramon, Ibiza: investigating diet and mobility through light stable isotopes in bone collagen and tooth enamel. *Archaeol. Anthropol. Sci.* **11**, 3913–3930 (2019).
115. López-Costas, O. & Müldner, G. Boom and bust at a medieval fishing port: dietary preferences of fishers and artisan families from Pontevedra (Galicia, NW Spain) during the Late Medieval and Early Modern Period. *Archaeol. Anthropol. Sci.* **11**, 3717–3731 (2019).
116. Inskip, S., Carroll, G., Waters-Rist, A. & López-Costas, O. Diet and food strategies in a southern al-Andalusian urban environment during Caliphal period, Écija, Sevilla. *Archaeol. Anthropol. Sci.* **11**, 3857–3874 (2019).
117. Martiniano, R. *et al.* The population genomics of archaeological transition in west Iberia: Investigation of ancient substructure using imputation and haplotype-based methods. *PLOS Genet.* **13**, e1006852; 10.1371/journal.pgen.1006852 (2017).
118. Olalde, I. *et al.* The Beaker phenomenon and the genomic transformation of northwest Europe. *Nature* **555**, 190–196 (2018).
119. Valdiosera, C. *et al.* Four millennia of Iberian biomolecular prehistory illustrate the impact of prehistoric migrations at the far end of Eurasia. *Proc. Natl. Acad. Sci.* 201717762 (2018) doi:10.1073/pnas.1717762115.
